# Synergy of photocatalysis and fuel cells: A chronological review on efficient designs, potential materials and emerging applications

**DOI:** 10.3389/fchem.2022.1038221

**Published:** 2022-11-25

**Authors:** Sangeeth John, Wojciech Nogala, Bhavana Gupta, Shubra Singh

**Affiliations:** ^1^ Crystal Growth Centre, Anna University, Chennai, Tamil Nadu, India; ^2^ Institute of Physical Chemistry, Polish Academy of Sciences, Warsaw, Poland

**Keywords:** photocatalytic fuel cells, hydrogen evolution (HE), CO_2_ reduction, heavy metal reduction, sensor, photocatalysis

## Abstract

The rising demand of energy and lack of clean water are two major concerns of modern world. Renewable energy sources are the only way out in order to provide energy in a sustainable manner for the ever-increasing demands of the society. A renewable energy source which can also provide clean water will be of immense interest and that is where Photocatalytic Fuel Cells (PFCs) exactly fit in. PFCs hold the ability to produce electric power with simultaneous photocatalytic degradation of pollutants on exposure to light. Different strategies, including conventional Photoelectrochemical cell design, have been technically upgraded to exploit the advantage of PFCs and to widen their applicability. Parallel to the research on design, researchers have put an immense effort into developing materials/composites for electrodes and their unique properties. The efficient strategies and potential materials have opened up a new horizon of applications for PFCs. Recent research reports reveal this persistently broadening arena which includes hydrogen and hydrogen peroxide generation, carbon dioxide and heavy metal reduction and even sensor applications. The review reported here consolidates all the aspects of various design strategies, materials and applications of PFCs. The review provides an overall understanding of PFC systems, which possess the potential to be a marvellous renewable source of energy with a handful of simultaneous applications. The review is a read to the scientific community and early researchers interested in working on PFC systems.

## 1 Introduction

The recent idea to explore renewable power sources has been crucial due to many vital environmental concerns of pollution and depletion of natural resources. Researchers have already understood the need for efficient mechanisms to restore the environment and to produce sustainable energy. In this context, photocatalysis has shown a tremendous impact with its compatibility to produce energy in the form of hydrogen fuel and to simultaneously degrade a wide variety of pollutants from polluted water sources. Consumption of natural solar irradiation and simplicity in design makes photocatalysis a promising candidate in arenas of green energy production and wastewater treatment (Roberta L and Wilson F, 2001; Mao et al., 2012; Zhao et al., 2015; Puga, 2016; Ayekoe et al., 2017; Miseki and Sayama, 2019).

Photocatalysis is a synergistic combination of photochemistry and catalysis (Saravanan et al., 2017). In this method, light energy and catalytic property are simultaneously used to enhance the rate of a reaction. Akira Fujishima and Honda pioneered an experiment on the photocatalytic splitting of water using TiO_2_ in 1972, which remains as the foundation work on photocatalysis (FUJISHIMA and HONDA, 1972). Subsequently, scientific fields have witnessed a steep leap on this frontier owing to its wide range of applications in hydrogen generation, air purification, carbon dioxide reduction, antibacterial activity and wastewater treatment. (Li and Li, 2001; Dong et al., 2014; Leyland et al., 2016; Kadam et al., 2019; Wang et al., 2020c). Photocatalytic process is garnering more attention on the grounds of wastewater treatment with its capability towards complete mineralization of the pollutant at room temperature and pressure (Malato et al., 1999; Rizzo et al., 2009; Bernabeu et al., 2011). The capability of photocatalysis to perform wasteless degradations and superior response towards natural solar irradiation unceasingly drives researchers to search for new horizons. Photocatalytic fuel cell (PFC), an improvised design of Photoelectrochemical cells (PECs), can be considered as a recent milestone along the way.

PECs are divided into three categories: Regenerative Solar Cells, Photosynthetic Cells and Photocatalytic Fuel Cells. O’Reagen and Gratezel hold the prime report for the regenerative solar cells, where the solar energy is converted into electrical energy (O’Regan and Grätzel, 1991). In photosynthetic cells, solar energy is transformed into solar fuels, mainly hydrogen. The above-mentioned work by Fujishima and Honda was the first report in photosynthetic cells, where hydrogen evolution reaction was steered through photocatalytic water splitting (FUJISHIMA and HONDA, 1972). The photocatalytic fuel cell (PFC), evolving from photoelectrochemical (PEC) cells, is a more promising technology for organic waste remediation, electricity generation, hydrogen production, CO_2_ reduction, H_2_O_2_ generation, sensor applications, heavy metal reduction *etc.* Even though the PEC and PFC share similarities on the grounds of design and mechanism, the functional capability of PFC to extract chemical energy from organic molecules, without external bias is its key strength over PEC. In PFC, photocatalytic activity leads to electricity generation with or without hydrogen evolution, using organics as a fuel. Even though there is no specific report which can be stated as the primary report on PFC due to its prolonged transformation from PEC, Kaneko et al. was the first group to coin the term Photo Fuel cells. The reported photo fuel cell used TiO_2_ and Pt as photoanode and dark cathode respectively and NH_3_ as pollutant or “fuel” for the cell (Kaneko et al., 2005). This work kick-started studies on PFC in 2005, and from then, numerous reports have been published worldwide. Appropriate studies on PFC systems with enhanced efficiency shall be considered as a flawless scenario to tackle the upcoming global issues of water pollution and energy scarcity.

Photocatalytic fuel cell (PFC) further carries forward the concept of photocatalysis by degradation of pollutants and simultaneous electricity generation, in some cases, along with hydrogen generation (Liu et al., 2011a; Seger et al., 2012; Li J. et al., 2013; Wu et al., 2015). PFCs provide a synergistic effect of complete mineralization of pollutants by photocatalysis and sustainable electricity generation mechanism of fuel cells. In spite of various modifications constantly coming up in the design and materials of PFC, the basic strategy and principle remains intact. The basic design of a PFC consists of electrodes and suitable electrolyte as in PEC, with a substrate to be used as fuel. The photoanode is a photocatalyst semiconductor and the dark cathode is an electrocatalyst of noble metal. The electrolyte is chosen based on its ionic characteristics and the electrodes. Fuel or substrate has a wide range to choose from, since the photocatalytic property has a non-selective nature towards degrading the organics (Ueno et al., 2009). When the photoanode is composed of an n-type photocatalytic semiconductor, it acts as a negative electrode since the Fermi level lies near the conduction band edge of the n-type semiconductor. The chemical reactions occurring on the cathode purely rely on the aeration provided in the cathode compartment. In the absence of oxygen, the cathode behaves as a hydrogen electrode, whose potential is 0 V (vs. reversible hydrogen electrode) and the hydrogen evolution reactions occur (Daneshvar et al., 2004). But in the presence of oxygen, it acts as an oxygen electrode, whose potential under acidic conditions is determined by the reactions:

$$O_{2}+4H^{+}+4e^{-}\to 2H_{2}O\left(+1.23\;V\right)$$ (1)

$$O_{2}+2H^{+}+2e^{-}\to H_{2}O_{2}\left(+0.68\;V\right)$$ (2)

When the light is irradiated on the photocatalyst in the anode section, photoexcited electron-hole pairs are generated. Due to the difference in the potential levels of anode and cathode, the generated voltage drives the electrons in the external circuit, producing electrical energy. Photogenerated holes in the anode compartment work on the degradation of fuel through the conversion of hydroxyl ions to hydroxyl radical, which is one of the most reactive oxygen species. The photogenerated electrons contribute to hydrogen evolution reactions at the cathode in the absence of oxygen. Ion transport membranes are used between the anode and cathode compartments to enhance the sustainability of the cell. Although this is a basic design of PFC, numerous alterations and studies have been done to improve the efficiency of the system. The plausible reactions at anodic and cathodic sites are given in Eqs 3-16, as follows:

At anode:

$$Photoanode+h\nu \;\left(light\right)\to h^{+}+e^{-}\left(photoanode\right)$$ (3)

$$h^{+}+H_{2}O\to \;•OH+H^{+}$$ (4)

OH+Pollutant→Less toxic degraded products/Intermediates (5)

$$h^{+}+Pollutant\to \;Less\;toxic\;degraded\;products/Intermediates$$ (6)

At cathode:

$$Photocathode+h\nu \;\left(light\right)\;\to \;h^{+}+e^{-}\;\left(photocathode\right)\;\left[only\;in\;photocathode\right]$$ (7)

$$O_{2}+4H^{+}+4e‾\to 2H_{2}O\;\left[aerobic\;condition\right]$$ (8)

$$2H^{+}+2e‾\to H_{2}\left[anaerobic\;and\;acidic\;condition\right]$$ (9)

$$2H_{2}O+2e‾\to 2OH‾+H_{2}\;\left[anaerobic\;and\;alkaline\;condition\right]$$ (10)

$$O_{2}+e‾\to O_{2}^{‾•}$$ (11)

$$O_{2}+2H^{+}+2e‾\to \;H_{2}O_{2}$$ (12)

$$O_{2}^{‾•}+H^{+}\to {HO}_{2}^{•}$$ (13)

$$•OH+H_{2}O_{2}\to {HO}_{2}^{•}+H_{2}O$$ (14)

$$O_{2}^{‾•}+Pollutant\;\to \;Less\;toxic\;degraded\;products/Intermediates$$ (15)

•OH+Pollutant→Less toxic degraded products/Intermediates (16)

The present review article concentrates on the wide spectrum of research reports available on PFC to help the scientific community to get detailed information on an interesting and important subject from the viewpoint of renewable energy. The first review article, published by Panagiotis Lianos in 2011, discussed the basic concepts of PFC and 6 years later, the group came up with a consolidated work dealing with designs of various PFC systems (Lianos, 2011, 2017). Advanced materials used as PFC photo-electrodes and their engineering techniques were thoroughly studied by Li et al., in 2019 (Li M. et al., 2019). Ermete Antolini presented a review on PFCs in 2019, which gives a collective study on various materials and properties of PFC (Antolini, 2019). The various materials for electrodes in a PFC and the application of Fenton reactions in PFC systems were combined and presented by Yasser et al. (Vasseghian et al., 2020). A detailed review on the PFC systems focusing on their structural insights, charge transport, thermodynamic behavior and challenges were presented by Priyanka et al. in their recent review article (Mishra et al., 2021). Yun He et al. recently reviewed the PFC research, where the fundamentals and technological advancements along with various cell configurations have been thoroughly discussed (He et al., 2022). Since researchers have merged peer reviewed reports on the basics, designs, materials and properties of PFC systems, it seems to be a perfect phase to shed light on less explored, yet booming applications of PFC. A detailed review containing all the possible information along with the applications of PFC should be a higher priority at present.

## 2 Developments in design of PFC

When compared to PECs, a PFC differs in the biasing provided in the system to have a sustainable photo-generated charge transfer. In PECs, external electric biasing is provided for efficient transfer of photo-generated charges either with a standard electric cell or using a PV system. The need for external biasing for competent performance in PECs makes them less preferable on system design, cost and energy efficiency. PFCs do not require a peripheral biasing because either one or both electrodes are capable of photo-induced charge generation and their suitable Fermi level difference provides the required biasing. The scavenging of holes by fuel enhances the charge separation and transfer in photoelectrodes. The synergistic effects of this particular concept have been applied in many designs throughout the years.

### 2.1 Single chamber single photo-electrode

**FIGURE 1:**
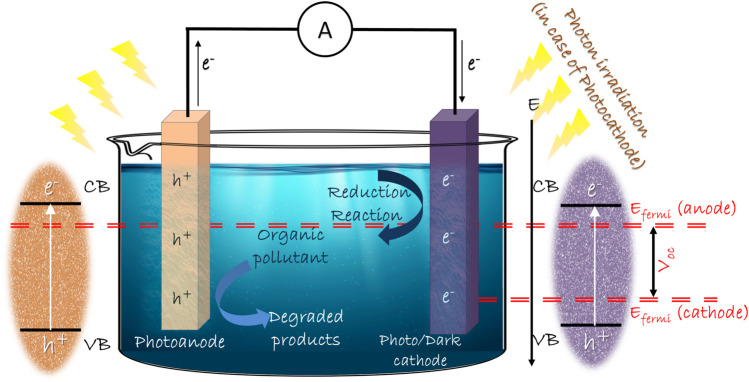
Schematic representation of Single Chamber PFC system design.

In this system, a photocatalytic semiconductor material and an electrocatalytic noble metal are employed as photo-anode and dark cathode, respectively, in a single chamber filled with electrolyte and fuel (Figure 1)*.* The first ever reported photo fuel cell by Kaneko et al. followed this design, utilizing film electrodes (Kaneko et al., 2005). In most reports, n-type photoanode is used together with dark cathode to establish a steady current flow (Ueno et al., 2009; Li K. et al., 2013; Lee et al., 2016a; Xie and Ouyang, 2017). TiO_2_ photoanode and Pt dark cathode is the maximum reported combination in SCSP systems, owing to the higher efficiency and superior photocatalytic performance of TiO_2_(Antoniadou et al., 2010; Li N. et al., 2018; Xie et al., 2019). The SCSP system gained a lot of attention with its minimalism in design and simple but effective overall reactions. In this system, the required bias for the efficient separation and transfer of charge carriers is primarily constituted by the band edge positions and type of photoanode. The influence of pH of the solution in the system is curtailed in this strategy. SCSP design also benefits in reducing the distance between anode and cathode, thus dropping the total resistance of the system. The cost-effectiveness of the overall design is commendable in SCSP since the design is free from ion-exchange membranes as seen in dual chamber systems.

The photoanode is irradiated with a light source which promotes the generation of electron-hole pairs. Later, these electron-hole pairs are separated due to the difference in Fermi level energy between electrodes. The electrons move towards the dark cathode through the external circuit, which renders useful electric power. At the same time, the holes attack the organic pollutants and degrade them to low pollution causing intermediates or non-toxic byproducts.

### 2.2 Single chamber dual photo-electrode

In SCSP design, the use of expensive noble-metal dark cathode is a significant disadvantage. Pt is widely used as a dark cathode because of its superior activity along with its non-toxic and non-corrosive nature (Zeng et al., 2018b; Li N. et al., 2019; Ong et al., 2019; Tang et al., 2019). SCDP design comprises a photoanode and photocathode in a single chamber containing electrolyte and substrate (Figure 1). It is capable of eliminating the convention of an expensive noble-metal cathode in SCSP systems. It also simplifies the system by eliminating the use of proton exchange membranes. Dual photoelectrodes help in trapping a wide spectrum of light irradiation and synergistically induce the degradation of pollutants along with electricity generation. The photoanode should be an n-type photoelectrode, which has a valence band with positive potential capable of pollutant/fuel oxidation and the photocathode can be a p-type photoelectrode, which has a conduction band with negative potential, for oxidant reduction. Moreover, the Fermi level of the photoanode should be at a higher potential than that of the photocathode for spontaneous current flow (Chen et al., 2012). Therefore, careful choice of n-type and p-type material can induce an internal bias between the photoelectrodes which can drive the electrons from anode to cathode. SCDP provides a single chamber setup for efficient utilization of photogenerated holes at photoanode and electrons at photocathode (Nahyoon et al., 2019a; Rabé et al., 2019b).

### 2.3 Dual chamber single photo-electrode

**FIGURE 2:**
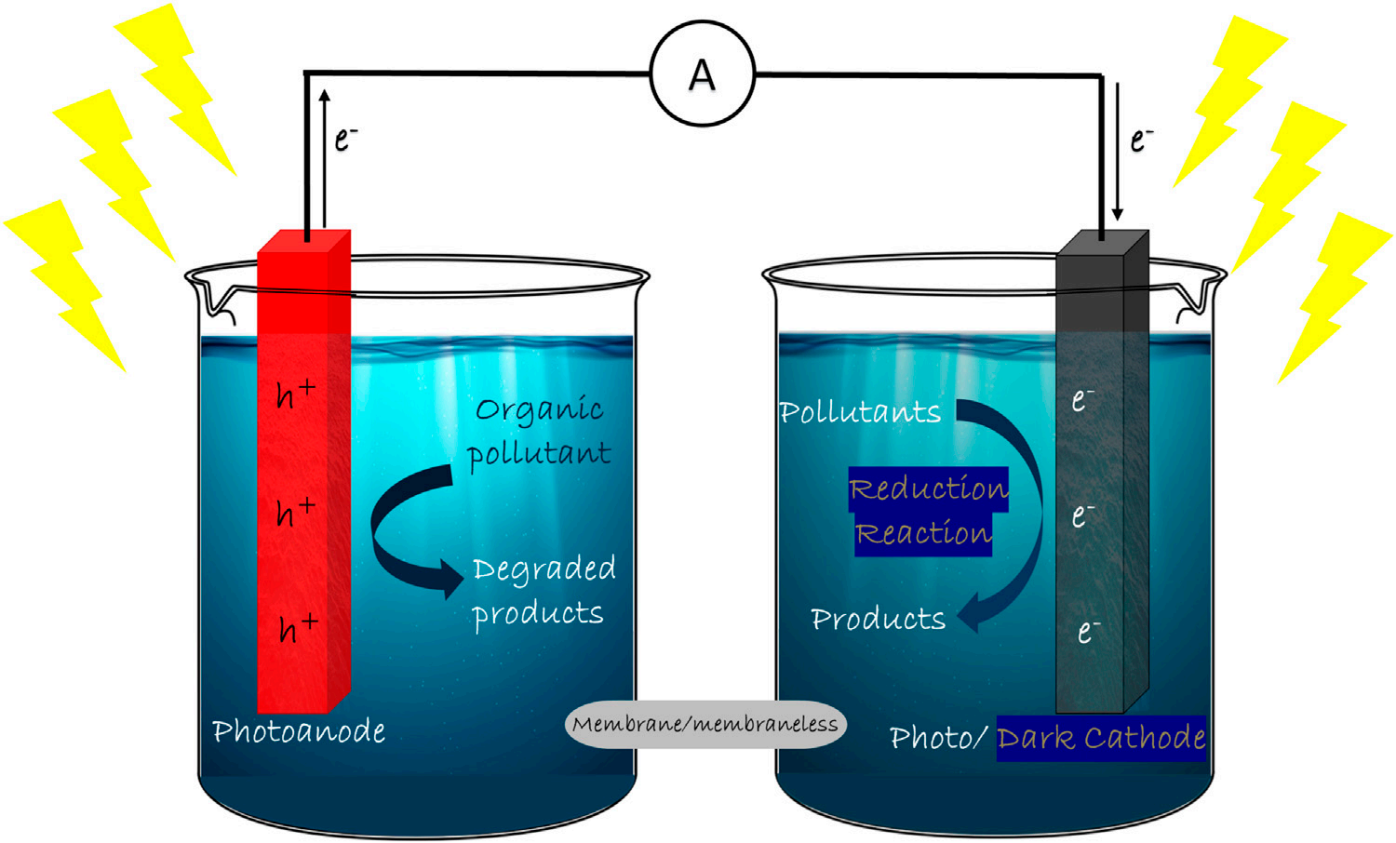
Schematic representation of Dual Chamber PFC system design.

In DCSP design, we have two distinct compartments, containing the same or different electrolytes, fuel in the photoanode compartment and oxidizer in the dark cathode compartment. In SCSP design, the internal biasing of the system was established by the photoelectrode and their respective band edge positions. In the DCSP system, the biasing depends on the photoelectrode and also the pH in both compartments. Even though DCSP design is more complicated and expensive involving the usage of proton exchange membrane, the wide range of combinations on photoelectrodes, electrolytes and substrates makes it a potential design to achieve proficient efficiency. The first report on DCSP designed cell for electricity generation and simultaneous hydrogen production utilizing ethanol, was by Maria Antoniadou in 2008 (Antoniadou et al., 2008). Among the dual chamber designs, the H-shaped reactor is the most widely used design (Figure 2). In DCSP, the light is irradiated on the anode site alone since it is the only photoactive electrode. The DCSP design has been effectively employed in electricity generation and simultaneous wastewater treatment. It was also recently employed for simultaneous dye degradation at the anode site and heavy metal removal at the cathode site, which hints towards the wide arena of applications this design can achieve. A wide variety of organic pollutants such as pharmaceutical drugs and dyes have been used as fuel to generate electricity using this design (Hu et al., 2015; Deng et al., 2018; Khalik et al., 2018; Pan et al., 2019).

### 2.4 Dual chamber dual photoelectrode system

In DCDP systems, the pros of employing dual photoelectrodes and advantages of dual chamber systems has been merged for better performance (Figure 2). The dual photoelectrodes can enhance the output voltage which will be the difference between quasi-Fermi levels of photoanode and photocathode. In this case, the electrons and holes generated in anode site will be utilized for electricity generation and degradation reaction, respectively. The photogenerated electrons of cathode side could react with oxygen to induce the oxygen reduction reaction (ORR), and photogenerated holes could be used to consume the electrons in the external circuit. Recently a formaldehyde PFC system was reported by Yanming Wang et al. where they have employed TiO_2_ nanorods (NRs) array as photoanode and Cu_2_O photocathode for formaldehyde oxidation. The micro-nano structure of the electrodes are controlled for high mobility of charge carriers, which resulted in a short-circuit current of 1.2 mA cm^−2^ and an open-circuit voltage of 0.58 V (Wang et al., 2020b). An improved short-circuit current density (J_sc_), the open-circuit voltage (V_oc_) and the maximum power output (P_max_) of 5.18 mA cm^−2^, 0.82 V and 0.91 mW cm^−2^ respectively was reported by BingzhiQian et al. The group designed a DCDP system as a methanol-based PFC which incorporated ZnO/BiVO_4_ photoanode and Cu_2_O photocathode. The interior potential difference between n-type ZnO/BiVO_4_ and p-type Cu_2_O accounts for the enhanced performance by actuating the electrons on the photoanode to transfer through the external circuit to photocathode, combining with the holes and generating electricity (Qian et al., 2020). Recently a visible-light-driven photocatalytic fuel cell (PFC) with DCDP design was employed, in which graphitic carbon nitride (g-C_3_N_4_) on W/WO_3_ nanorod arrays (W/WNR/g-C_3_N_4_) was used as the photoanode and Fe^3+^-doped CuBi_2_O_4_ thin film on indium tin oxide (ITO) conductive glass (ITO/CBFeO) was used as the photocathode. The one-dimensional structure of photoanode and the Fermi level mismatch between electrodes improved the generation and transfer of charge carriers. The short circuit current and maximum power density could reach 620 μA cm^−2^ and 110 μW cm^−2^, respectively (Huang et al., 2022).

### 2.5 Triple chamber system

**FIGURE 3:**
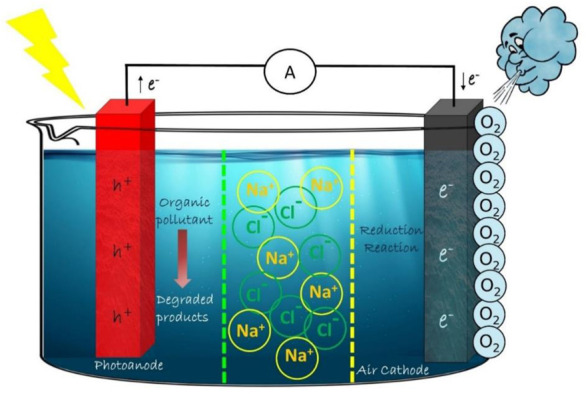
Schematic representation of Triple Chamber PFC system.

Several improvements have been brought in PFC designs to arrive at a stable system with improved efficiency. To enhance the charge separation in photoanode, many techniques have been employed in the system, such as engineering on electrode materials, anodic biasing *etc.* Providing a bias voltage at the anode site resulted in an improved degradation of organics, but a poor performance on the electricity generation. Sui et al., in 2015 came up with a triple chamber system, which makes use of salinity gradient power (SGP) to enhance the charge separation (Figure 3). The revocable mixing of solutions with high and low salt concentrations produces SGP (Veerman et al., 2010; Tedesco et al., 2015).

Usually, the degradation of the fuel reduces the competent charge separation and hence the current. But the triple chamber system using SGP was able to produce a stable current for a long time. The ability of chloride ions to get oxidized to chlorate or perchlorate by holes helped the salt solution to play the role of a substitute fuel which accounted for the stable current in the system (Sui et al., 2015).

### 2.6 Micro fuel cells

The relatively large distance between electrodes in PFC designs increases the mass transfer resistance and hence accounts for a decline in the efficiency of the whole system. A membrane between the chambers for ion transport, which facilitates the continuity of the system, was used in PFCs, which boosted the cost of conventional designs. Another major drawback is that the specific reaction surface is small, which limits cell performance. The design of PFCs always aimed at overcoming these drawbacks. The first such attempt was made by Li et al., in 2014 in the form of Optofluidic Micro Fuel Cells (Li et al., 2014).

Optofluidics provide the synergistic advantages of optics and microfluidics, such as fine flow control, enhanced surface to volume ratio and uniform light distribution (Psaltis et al., 2006; Erickson et al., 2011). Optofluidic-based design also helps in the reduction of distance between the electrodes and elimination of membranes. The first optofluidic PFC proposed by Li et al. had a stacked arrangement of electrodes and employed TiO_2_ and Pt as photoanode and cathode, respectively (Li et al., 2014). A modification of this design was proposed in 2016 by implementing a shoulder-to-shoulder arrangement of electrodes where the electrode materials remained intact. Uniform utilization of light irradiation was coined as a major advantage for this design (Xia M. et al., 2016). Chen et al., in 2018 took the MFC strategy to the next level by using a visible light responsive photoanode without any change in the basic design (Chen et al., 2018). Recently Liu et al. have reported an MFC which employed a photoanode and photocathode with a similar design as reported by Chen et al. Dual photoelectrode MFC have advantages of both MFCs and dual photoelectrode systems as we have explained above (Liu J. et al., 2019). Dual photoelectrode optofluidic fuel cell in which the usage of precious metal Pt electrode is avoided with a photocathode has been recently reported by Yun He et al. The cell consisted of Cu_2_O photocathode and a BiVO_4_ photoanode placed shoulder-to-shoulder in a microchamber, offered a large contact area between electrolyte and photoelectrodes. The photoelectricity conversion in using glucose resulted in open-circuit voltage and short-circuit photocurrent of 0.463 ​V and 0.113 ​mA cm^−2^, respectively (He et al., 2021).

### 2.7 Rotating disc PFC

Rotating disc reactor design has been proposed by many groups as an improved strategy among photoelectrochemical cells. Rotating disc design renders a synergistic attribute of highly efficient thin-film PEC design (in which the upper half of the rotating disk photoanode with a thin layer of pollutant solution is exposed to light radiation in air) and the conventional PEC design (in which the other half of the disk is submerged in bulk pollutant solution), irradiated by the same light source. The schematic design of the RDPFC system can be depicted as in Figure 2 with a modification of photoanode with rotating disc photoanode. The quantum yield of photogenerated charge carriers in the thin film half of the rotating disc design will be high due to enhanced interaction with light radiation. Hence there will be efficient oxidation of organic compounds. This thin film is unceasingly restored by the rotatory motion of the electrode, which also facilitates the mass transfer of pollutants and degradation products. Combining this thin film design with conventional bulk reactor design rotating disc design opens up a wider arena of innovative design (Xu et al., 2008, 2009).

Rotating disc design was adopted into photo fuel cells by Tang et al., in 2014, where TiO_2_ rotating disc electrode was utilized to degrade Rhodamine B(Rh B) and reactive brilliant red X-3B (Tang et al., 2014). It was shown that the adaptation of RD design decupled the hydrogen generation rate and electric flow compared with conventional design. A polypyrrole-based cathode was established on RD design by Li et al., in 2016 (Li K. et al., 2016). Later the RD design was further modified by Jun Zhang et al., in 2019 as they incorporated multiple rotating disc cathodes in PFC. In the multi cathode RD design, they succeeded in achieving a P_max_ of 0.22 mW, which was 53% greater than a single cathode design. The obtained current, power and degradation efficiency values of RDPFCs reveal that the future of this design is appealing, even though the implementation is a bit complicated (Zhang et al., 2019c; 2019b).

A thin-film rotating disk photocatalytic fuel cell (PFC) reactor employed for enhanced photocatalytic reduction of CO_2_ to CH_4_. The yield of CH_4_ in this system was measured to be 7.62 μmol g^−1^ h^−1^. Diffusion of CO_2_ through the thin film and high solubility of CO_2_ in electrolyte enabled effective utilization of CO_2_ and improved conversion efficacy (Zhang et al., 2020c). A recent report was published by the same group on the gaseous formaldehyde removal using a RDPFC design with TiO_2_ photoanode and Pt cathode. Rotating disc design improved the efficiency of the system with its advantages of greater absorption, fast oxidation and humidity less sensitivity (Zhang et al., 2020d).

Numerous parameters affect the overall efficiency of a PFC system. Even though the efficacy of the PFC design is the major factor to look for, overall cost, simplicity, scalability and application are the other entities which play vital role. Table 1 provides a summary of the pros and cons of various designs.

**TABLE 1:** Pros and cons of various PFC designs.

Sl. No:	Design of PFC	Advantages	Disadvantages
1	Single Chamber Single Photoelectrode	• Simple Design	• Choice of Photoanode is limited
		• No need of PEM, hence the overall cost is reduced	• Overall cost moves to higher side when Pt is used
		• Minimum distance between electrodes reduces the overall resistance	• Single chamber limits the use of multiple organic pollutants
2	Single Chamber Dual Photoelectrode	• Replacing Pt with photocathode reduces the overall cost	• The selection of proper combination of electrodes is delicate • Single chamber limits the use of multiple organic pollutants
		• No need to use PEM	
		• Low resistance	
3	Dual Chamber Single Photoelectrode	• Different contaminant degradation or various applications can be performed simultaneously	• Overall cost moves to higher side when Pt and/or PEM is used in the system
		• Biasing can be introduced and controlled by pH variation in chamber	
4	Dual Chamber Dual Photoelectrode	• Different applications in each electrode site	• Overall cost moves to higher side PEM is used in the system. • Complex design, as compared to single chamber design
		• No need of Pt cathode	
		• Biasing can be introduced and controlled synergistically by the pH of the chambers and the fermi level mismatch of the photoelectrodes	
5	Triple Chamber Design	• SGP can provide stable current for a long time	• Multifaceted design
6	Micro Fuel Cell Design	• Low resistance, since the distance between anode and cathode is minimum	• Complex micro-fabrication techniques • Mostly suitable for Sensor applications rather than mass pollutant degradation and power generation
		• Elimination of membrane	
		• Uniform utilization of light irradiation	
7	Rotating Disc Design	• Synergy of thin film and PEC design	• Complex design along with motorized rotating photoelectrode
		• Enhanced efficiency by utilizing continuously rotating photoelectrode	

### 2.8 Hybrid systems and novel designs

**FIGURE 4:**
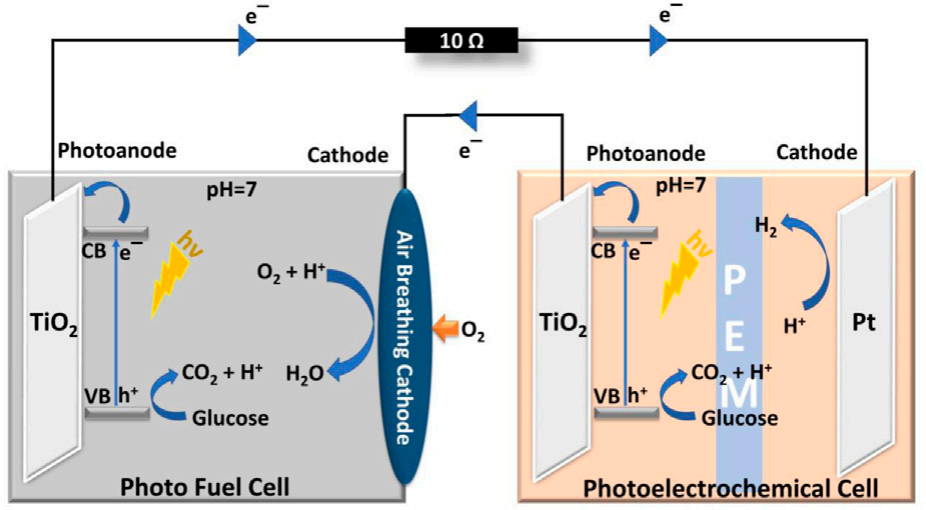
Hybrid system incorporating PFC and PEC. Reproduced with permission (Liu et al., 2017), Copyright 2022, Elsevier.

Recent progress on PFC design includes exploring the combination of conventional PFC designs with other well-explored systems and have been discussed here under the hybrid category. Hybrid systems are expected to enhance the efficiency of a single system in order to widen the applicability of the design. The first reported hybrid system combined the conventional PFC design with a Fenton reactor to efficiently degrade Reactive Black five and simultaneously produce electricity. The design incorporated an SCSP PFC design with RB5 dye and a Fenton reactor to produce better degradation efficiency and current generation than these systems separately (Nordin et al., 2017). PFC was used as a potential source for efficient working of PEC in the hybrid system proposed by Liu et al. In this PFC-PEC hybrid setup, the chemical energy of glucose is converted into electrical energy by an SCSP PFC and this electrical energy was utilized as a potential bias for the PEC reactor. TiO_2_ and Pt were employed as photoelectrodes in both cells, as shown in Figure 4. The synergy of the hybrid system produced an increased photocurrent and a commendable hydrogen evolution rate (Liu et al., 2017). A photovoltaic system pooled with PFC design was introduced by Zeng et al., where the group has demonstrated a self-sustaining monolithic photoelectrocatalytic/photovoltaic system (SMPP system). In this SMPP system, the photoanode comprising of WO_3_/BiVO_4_ was attached with a Si-PVC, which enhanced the absorption wavelength range of the system to produce a power density of 1,112 μW cm^−2^, 14 times greater than the conventional PFC reported back then.

The group have recently constructed an unassisted, hybrid tandem photocatalytic fuel cell (HTPFC) constructed by adhering a silicon solar cell (SSC) to the back of a highly-active silicon-doped TiO_2_ nanorod array (STNR) for efficient solar hydrogen production (28.8 μmol h^−1^ cm^−2^) coupled with tetracycline degradation (Zeng et al., 2018b) (Zeng et al., 2020). If the above hybrid systems focused on enhancing the efficiency of separate systems by coupling them, some systems focused on widening the applicability of the same. A PFC-PVC system with a Pd-Cu modified Ni foam cathode was capable of removing total organic carbon (TOC) and total nitrogen from wastewater with simultaneous electricity generation (Zhang Y. et al., 2018).

**FIGURE 5:**
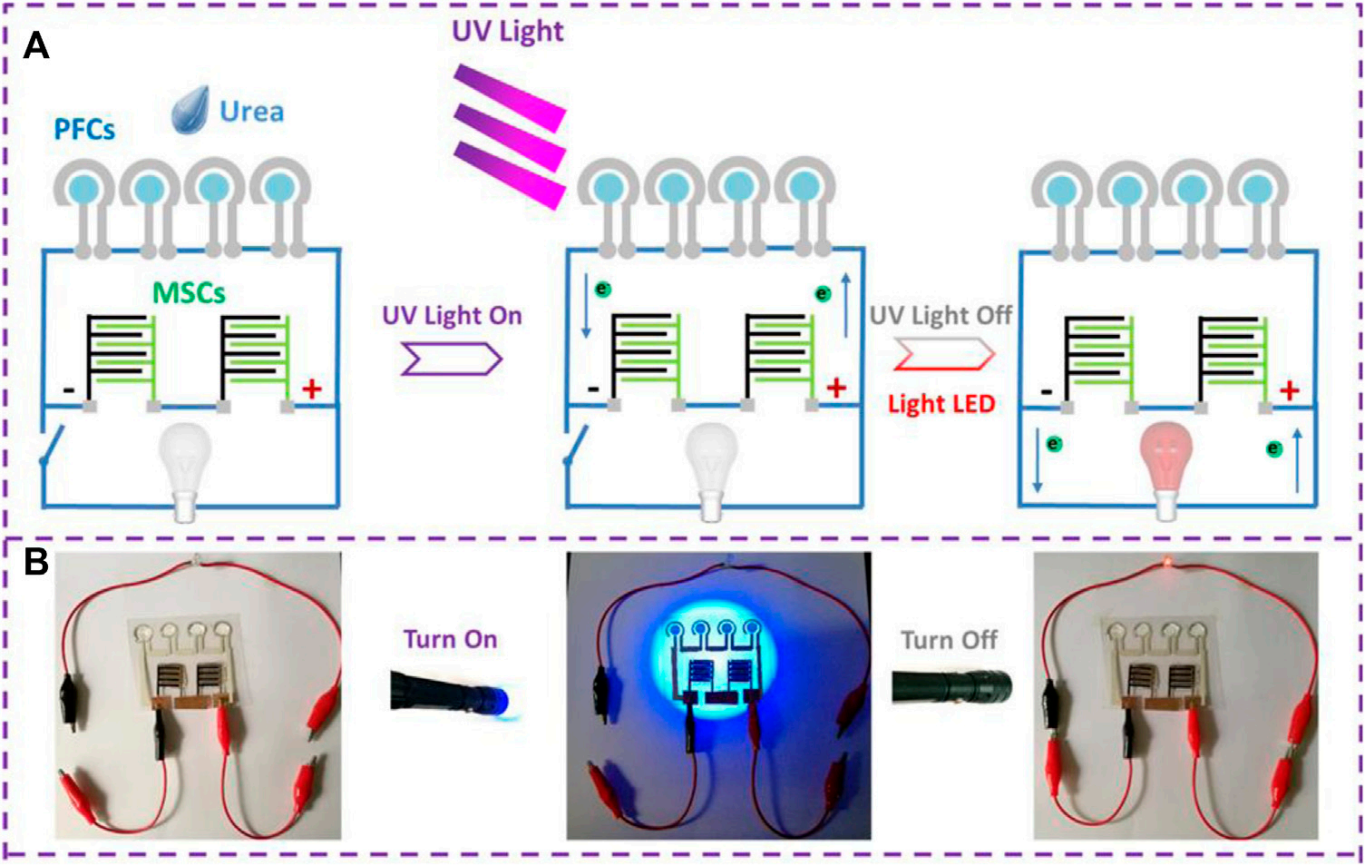
**(A)** Schematic diagram of self-powered system including in-plane PFCs and MSCs for powering red LED **(B)** real time photographs depicting four tandem planar PFCs charging two Micro Supercapacitors in series, which later power a red LED. Reproduced with permission (Qiu et al., 2019), Copyright 2019, American Chemical Society.

Jiao et al. reported an innovative combination of CO_2_ photoreduction system with PFC successfully generating electricity. The 3D TiO_2_-Ni foam was used to reduce CO_2_ into hydrocarbons and this was fed into PFC as a fuel to produce electric power (Jiao et al., 2019). A step further on the design was achieved by Meijia Qiu et al. They reported a PFC combined with an Asymmetric Micro supercapacitor (AMSC), which can not only generate electric power simultaneously with wastewater degradation but also can store energy to power small portable electronics (Figure 5). TiO_2_ was employed as photoelectrode, Ag as counter electrode and urea as fuel to achieve a maximum power density of 3.04 μW cm^−2^ (Qiu et al., 2019). Another interesting hybrid system was reported recently by Peng Xu et al., where the PFC system was combined with Reverse Electro-Dialysis (RED). The stacked arrangement of cation exchange membranes (CEM) and anion exchange membranes (AEM) provides a salinity gradient to drive the RED process for wastewater treatment. The group has reported an H_2_O_2_ production and obtained a maximum power of 76 W m^−2^ (Xu et al., 2019b; 2019c; Xu and Xu, 2019). A visible light active PFC integrating electro-Fenton (EF) process has been recently reported employing rGO/BiO_1−x_I photoanode and biomass-derived N-doped carbon (BNC) cathode. The system has a maximum power density (P_max_) of 17.50 μW cm^−2^ with formic acid as fuel (Hu et al., 2020). A similar PFC-EF hybrid system constructed with ZnO/C photoanode and carbon cathode for enhanced power generation and Amaranth dye degradation by Shen Hui Thor et al. (Thor et al., 2020). The effect of aeration and pH on the overall efficiency of the PFC-EF system has been thoroughly studied. Dual chamber setup is employed in which, one chamber consists of PFC and the later contains the electrodes for electro Fenton reaction. The system attained a maximum power density of 2.221 μWcm^−2^ and maximum current density of 0.012 mAcm^−2^. Nordin et al. developed a hybrid electrochemical system of photocatalytic fuel cell - peroxi-coagulation (PFC-PC)which involve enhanced hydroxyl radical formation for simultaneous degradation of organic pollutant and electricity generation (Nordin et al., 2020). Furthermore, a novel design of external loop airlift photocatalytic fuel cell (APFC) has been reported by Ammar et al. The cell contains Fe@CdS and g-C_3_N_4_ immobilized on a polystyrene film as photoanode and rGO/carbon brush was used as aerated cathode. The design consists of a riser section and downcomer section. In riser section air pumping is provided for the pollutant to rise up and through the photocathode brush. Photoanode is placed as the walls of downcomer tube. The APFC design rendered maximum power density (P_max_), open circuit voltage (V_oc_) and short circuit current density (J_sc_) were respectively 1.57 mW cm^−2^, 1.19 V and 2.525 mA cm^−2^ along with phenol degradation (Ammar et al., 2020). All of the above systems which utilizes or intend to utilize natural solar irradiation faces the major challenge of rendering continuous output throughout the day. A recent report which intends to tackle this issue is published by Zhang et al., which depicts a hybrid PFC system. The group developed a green supercapacitor assisted PFC system with a chemical bias, which aimed for storing photoelectrons by supercapacitors under light irradiation and discharging for hydrogen production under dark to enable continuous hydrogen production. The report put forward an alternative approach to achieve continuous H_2_ production, electricity generation and waste degradation using a supercapacitor coupled PFC system (Zhang et al., 2021).

The hybrid systems reported so far broadly open up the doors to an incredible future of PFC and its numerous applications.

## 3 Advancements in materials

Materials play a vital role in almost all scientific experimental research areas. The progress in materials science has already revealed the importance of proper choice and engineering of materials based on their property. As in every other field, research based on PFCs took a great leap forward by exploring the structure-properties correlation of various materials.

### 3.1 Materials for anode

The photoanode of a PFC determines the power generation and degradation of organic pollutants or fuels. Hence, the development of an efficient anode material greatly determines the overall efficiency of the PFC system. In spite of extensive exploration in this field, TiO_2_ still remains the champion material and has been reported as a photoanode in about 70% of the total reports on PFC. Chemical inertness over wide environments, ease of synthesizing, efficient thin film deposition, availability at low-cost and non-toxic nature holds TiO_2_ in the prime position as a photoanode (Kaneko et al., 2005; Balis et al., 2012; Seger et al., 2012; Ogura et al., 2014; Xia M. et al., 2016; Jiao et al., 2019). However, the wide bandgap of TiO_2_ allows it to be active only in the ultraviolet region of the photo-spectrum and it exhibits a higher electron-hole recombination rate. To overcome these drawbacks, various approaches, such as metallic and non-metallic doping, coupling with low bandgap semiconductors and morphological variations, have been explored. All anode materials reported other than TiO_2_ have been presented in Table 2.

**TABLE 2:** Quick view on reports of PFC employing anode materials other than TiO_2_.

Anode	Cathode	Electrolyte	Lamp	Organic pollutant/compound	V_oc_ (V)	J_sc_ (mAcm^−2^)	FF	References
BiOCl/Ti	Pt	Na_2_SO_4_	UV	RhB	0.0116	0.655	0.39	Li et al. (2013b)
			Visible	Methylene Blue	0.0063	0.585	0.19	
			Sunlight	Rh B	0.0055	0.648	0.21	
			Artificial Sunlight	Basic Orange	0.0262	0.646	0.23	
Ag-BiOI microsphere	Pt	Na_2_SO_4_	Visible Light	BPA	0.67	0.0258	0.32	Shu et al. (2014)
WO_3_/W nanopore structure	Pt/PVC	Na_2_SO_4_	Visible Light	Methylene Blue	0.38	0.350	-	Chen et al. (2014)
WO_3_ nanosheets	Pt/FTO	Na_2_SO_4_	Xenon Lamp	Methanol	0.52	0.06	-	Hu et al. (2015)
Nb_2_O_5_ hierarchical nanoflowers					0.96	0.02		
BiVO_4_	Pt/C	Sodium Borate	Xe Lamp	Glucose	0.76	2.28	-	Zhang et al. (2015b)
W- BiVO_4_			AM 1.5G	Glycerol	0.93	1.65		
BiVO_4_/WO3	Pt/BJS	KH_2_PO_4_	Solar Simulator	Phenol	0.81	0.43	-	Xia et al. (2016a)
ZnO/Zn rod like structure	Pt/C	Na_2_SO_4_	UV Light	Reactive Green 19	1.128	2.64	0.43	Lee et al. (2016b)
ZnO/Zn rod like structure	Pt/C	-	UV light	Reactive Green 19	-	0.0427	-	Lee et al. (2017)
WO_3_/FTO	Pt/CC	Na_2_SO_4_	Solar Simulator	Oxytetracycline Hydrochloride	0.45	0.372	0.21	Xie and Ouyang, (2017)
BiOCl {010} nanosheets	Pt	Na_2_SO_4_	Xe lamp	RhB	0.689	0.0058	0.11	Zhang et al. (2017b)
ZnO/Zn rod like structure	CuO/Cu	Na_2_SO_4_	UV lamp	Methyl Green	0.919	0.1033	-	Kee et al. (2018)
ZnO	Pt	-	Sunlight	RB5	1	0.0069	0.34	Khalik et al. (2018)
ZnO	Pt	-	UV lamp	Reactive Green 19	-	0.0041	-	Lee et al. (2018b)
ZnO/Zn	Pt	-	Sunlight	Reactive Green 19	0.704	0.0126	0.29	Lee et al. (2018a)
			UV lamp	Reactive Green 19	0.814	0.0101	0.26	
BiVO_4_ network morphology	CuO/Cu_2_O	Na_2_SO_4_	Visible Light	Phenol	-	0.30	-	Li et al. (2018b)
Fe-GO-TiP	ZnIn_2_S_4_	Na_2_SO_4_	Visible Light	RhB	0.4	-	-	Nahyoon et al. (2018)
BiVO_4_{040}	Pt	Na_2_SO_4_+Fe-EDTA	Xe lamp	Nicotinic Acid	1.13	1.37	-	Xia et al. (2018)
BiVO_4_/WO_3_	Pt	Na_2_SO_4_	Solar Simulator	Glucose	-	0.091	-	Xie et al. (2018)
Au-C_3_N_4_ nanosheet	Hemin-Graphene-	-	Visible Light	H_2_O_2_	0.45	-	-	Yan et al. (2018b)
TiO_2_/WO3/W nanorods, nanothorns	Pt-BJS	K_2_SO_4_	Solar Simulator	Atrazine	0.657	0.22	0.19	Zeng et al. (2018a)
WO_3_ nanoplates/BiVO_4_ -Si PVC	Pt	Na_2_SO_4_	Solar Simulator	Tetracycline Hydrochloride	1.35	0.29	-	Zeng et al. (2018b)
WO_3_/Si-PVC	Pd-Cu	Synthetic Wastewater	Solar Simulator	-	3.44	0.74	0.30	Zhang et al. (2018b)
BiOCl (001) Ultrathin Nanosheet	Pt	Na_2_SO_4_	Visible Light	Rh B	0.453	0.00391	0.30	Zhang et al. (2018a)
Fe_2_O_3_/BiVO_4_-PVC	Pt	Na_2_SO_4_/Fe^2+^/Tetra polyphosphate	Solar Simulator	Congo Red	2.75	1.66	-	Li et al. (2019a)
WO_3_/W	Pt	Na_2_SO_4_	Visible light	Methanol	0.180	0.0186	0.25	Pan et al. (2019)
Ag/Cr-BiOCl	Pt	Na_2_SO_4_	Visible Light	Methyl Orange	0.563	0.0008	0.43	Zhang et al. (2019d)
Fe/GTiP	ZnIn_2_S_4_	-	Visible Light	Berberine Chloride	0.65	-	-	Nahyoon et al. (2019b)
ZnO/Zn	FeVO_4_	NaCl	UV lamp	-	1.52	0.95	-	Xu et al. (2019b)
WO_3_/W	Fe@Fe_2_O_3_	Na_2_SO_4_	Visible Light	Methyl Blue	1.26	0.59	-	Xu et al. (2019a)
ZnO/Zn	FeVO_4_	NaCl	UV lamp	Coal gasification water	-	0.0162	-	Xu et al. (2019c)
Ag/ZnO/NiF	CoFe_2_O_4_	Na_2_SO_4_ + PMS	Visible Light	Berberine	0.625	0.165	-	Zhang et al. (2019e)
rGO/BiO_1−x_I	BNC	Na_2_SO_4_+ FeSO_4_	Visible Light (Xe)	Formic acid, 4-NP, BPA, salicylic acid solutions		0.133		Hu et al. (2020)

Degradation of organic substances facilitating electricity generation with CdS functionalized TiO_2_ was reported by Antoniadou et al., in 2009. This report can be considered as the first report on visible light activated PFC and was successfully accomplished by CdS functionalization (Antoniadou and Lianos, 2009). Heterostructured sulfide-TiO_2_ photoanodes have also been explored to enhance the absorption spectrum and to enhance the electron-hole separation. Many narrow bandgap semiconductors (other than sulfides) with energy band edge positions compatible with TiO_2_ have also been widely explored for fabricating a photoanode for PFC(Sfaelou et al., 2012; Wang et al., 2014; Liao et al., 2015; He et al., 2018; Kee et al., 2018; Raptis et al., 2018; Liu X. H. et al., 2019). Cu_2_O functionalized TiO_2_ was fabricated to enhance the current density and power density, as reported by Liu et al. (Liu et al., 2011a). Heterostructure photoanode, WO_3_-TiO_2_, was also proposed by some groups to enhance the overall efficiency of the PFC system by absorbing more photons and reducing the recombination rate. In a report by Zeng et al., TiO_2_ has been employed as a coating onto WO_3_ to overcome its drawbacks, such as sluggish charge transfer, gradual loss of photoactivity due to the formation of peroxo-species on its surface as well as rapid charge recombination due to surface defects (Hill and Choi, 2012; Kim et al., 2013; Yang et al., 2014; Zeng et al., 2018a). BiVO_4_-TiO_2_ heterostructures and ZnFe_2_O_4_-TiO_2_ photoanodes have also been explored to enhance light absorption, obtain maximum electron-hole separation and synergistically achieve stability (Bai et al., 2016; Xie et al., 2017; Lu et al., 2019).

**FIGURE 6:**
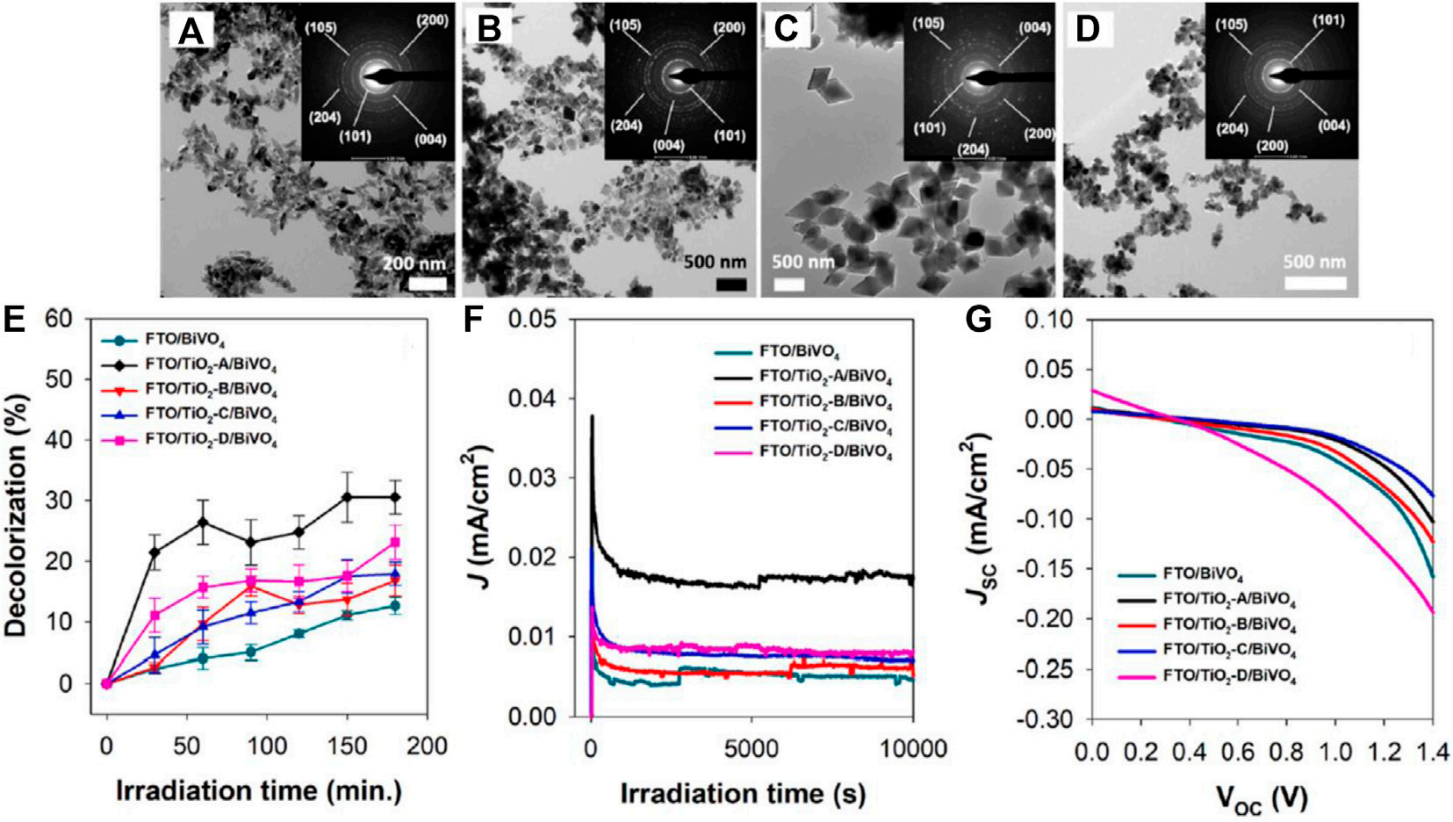
**(A–D)** TEM images (inset: SAED) of the as-prepared TiO_2_ nano spindles (TiO_2_-A), nano cubes (TiO_2_-B), nano-octahedra (TiO_2_-C), and nano-truncated octahedra (TiO_2_-D), respectively. **(E–G)** Performance of the PFC system in decolorization of RhB; J-t curve (measured at 0.8 V vs NHE), and J_SC_-V_OC_ curve of the PFC system in the presence of RhB and Na_2_SO_4_ using different types of photoanodes under light irradiation. Reproduced with permission (Khalil et al., 2021), Copyright 2021, Elsevier.

Morphological variants of many materials have shown improved properties compared with their pristine powder or bulk form. As we observe down the timeline of PFC photoanode development, we can see a majority of works concentrated on one-dimensional structures of materials like nanotubes, nanorods, nanowires, *etc.* One-dimensional structured photoanodes with increased surface area and reduced internal resistance possess many advantages like faster electron transport rate, enhanced photon absorption rate and improved organic pollutant adsorption. In this regard, besides TiO_2,_ which has been well explored for its one-dimensional structures, ZnO-based nanostructures have also been studied. (Liu et al., 2011b; Li J. et al., 2013; Li et al., 2016 X.; Ying et al., 2016; Zhang et al., 2019b) (Liu et al., 2011a; Li J. et al., 2013; Liao et al., 2015). Leaves-like CdS with a surface decorated by Pt-Co_x_ nanoparticles is employed as a photoanode to improve the photocatalytic hydrogen generation. The morphological variant showed improved H_2_ generation (45.09 mmol h^−1^g^−1^) compared to the particles (Jia et al., 2020). The effect of co-exposed TiO_2_’s (001) and (101) facets on the performance of TiO_2_/BiVO_4_ photoanodes in the PFC system has been successfully studied recently (Figure 6). Nano spindles, nano cubes, nano octahedra, and nano truncated octahedra of TiO_2_ has been synthesized to achieve the exposure of (001) and (101) facets. The active facet morphology of TiO_2_ (nano spindles and nano truncated octahedra) and BiVO_4_ is combined to form type-II heterojunction, which enhanced the overall photocatalytic efficacy (Khalil et al., 2021).

BiOCl/Ti, BiOCl facet modified anode candidates have been proposed and showed impressive results (Li K. et al., 2013). Detailed review on BiOCl facet engineered photoanode is shown in the review by Li et al., in 2019 (Li M. et al., 2019). Nb_2_O_5_, which has a similar bandgap, but a more negative conduction band edge than TiO_2_, has been compared with TiO_2_ and WO_3_ in work by Hu et al. TiO_2_ with its impressive internal quantum yield outperformed Nb_2_O_5_ in this work (Hu et al., 2015). ZnO, which has a similar bandgap and similar bandedge positions as TiO_2,_ has been an attractive material for photoanode fabrication with its remarkable photoactivity. However, the vulnerable nature towards oxidation limits its application (Lee et al., 2016b, 2017; Khalik et al., 2016; Kee et al., 2018; Lam et al., 2019; Ong et al., 2019). As a medium bandgap semiconductor, many have worked on BiVO_4_, which has been utilized as a visible light active photoanode. BiVO_4_ has also been combined with other metal oxide semiconductor materials for application in PFC(Zhang et al., 2015a; Zhang et al., 2017 B.; Xia L. et al., 2016; Zeng et al., 2018b; Xie et al., 2018; Lu et al., 2019). A hybrid EF-PFC system was recently reported to introduce an oxygen vacancies-rich rGO/BiOI (rGO/BiO_1−x_I) photoanode, which possess the advantages of excellent light-harvesting ability, superior charge carriers separation and transfer performance (Hu et al., 2020). Similar advantages are achieved by Z-scheme heterostructured photoanode Ag_3_PO_4_@g-C_3_N_4_, which ensured an effective separation of charge carriers to achieve a good PFC performance and also maintained a high redox ability to contribute the degradation of organics (Yu et al., 2020). Another novel Z-scheme photoanode WO_3_/ZnO/Zn has also been recently reported for photodegradation of sunset yellow and phenol (Lam et al., 2020).

Graphitic carbon nitride is a promising material with its visible light activity, tunable bandgap and low cost. The severe charge recombination in the materials limits its wide applicability in the photocatalytic regime. Numerous methods have been reported to reduce its charge recombination and thus enhance its efficiency. Boron doped g-C_3_N_4_ synthesized *via* addition of H_3_BO_3_ in urea varied the nanostructure and enhanced its activity towards photocatalytic hydrogen generation. Another report revealed synthesis of g-C_3_N_4_ by addition of cyano groups on its surface, which enhances the charge separation and transport in g-C_3_N_4_, which again enhanced its performance in hydrogen evolution reaction (Wang X. et al., 2021; 2022a; 2022c). Another visible active materials BiVO_4_ has been widely studied owing to its suitable band edge positions, stability and low cost. But the use of BiVO_4_ is limited due to its short hole diffusion length and poor electron transport properties. Many works have been reported to overcome this drawback and make use of BiVO_4_ as a photoanode in PEC and PFC systems. Songcan Wang and his group recently reported a MoO_3-x_/BiVO_4_ photoanode, where the oxygen vacancies in MoO_3-x_ improved the electron mobility in the heterostructure (Wang S. et al., 2020; Wang et al., 2021 S.; 2022b). A novel hybrid photoanode recently reported by a few groups incorporates a PVC with conventional photoanode. Zeng et al. introduced a WO_3_/BiVO_4_—Si PVC photoanode and have designed a self-sustaining monolithic photoelectrocatalytic/photovoltaic (SMPP) system, which efficiently degraded tetracycline hydrochloride with a commendable current and hydrogen evolution (Zeng et al., 2018b). TOC and TN were removed by Zhang et al., in 2018 by employing a WO_3_-Si PVC photoanode (Zhang Y. et al., 2018). Recently Li et al. have come up with a BiVO_4_/Fe_2_O_3_-Si PVC photoanode in PFC, using MB as a model pollutant. With an additional bias voltage, the generation of superoxide radical could be enhanced by 9 times. The maximum power density of the system achieved an appreciable leap owing to the enhanced ROS generation (Li L. et al., 2019). The hybrid photoanodes enable wider bandgap absorption and additional bias voltage for enhanced generation of ROS along with an improved PFC efficiency.

### 3.2 Materials for cathode

As compared to anode materials, there has been a dearth of research on cathode materials and their influence on PFC. Single photoelectrode systems (SCSP and DCSP) occupy more space in the timeline of PFC research and hence the Pt-based cathode materials were commonly employed. Due to the major drawback of Pt around the concern of cost and low radical generation efficiency, many photocathode materials like Buckypaper, Cu_2_O, ZnO, ZnIn_2_S_4_, WO_3_, FeVO_4_, and CoFe_2_O_4,_ *etc.* Were also explored by the researchers (Chen et al., 2012; Sfaelou et al., 2012; Bai et al., 2016; Liu et al., 2016; Nahyoon et al., 2019a; Rabé et al., 2019a; 2019b; Xu et al., 2019a; Zhang Y. et al., 2019; Wang et al., 2019; Xu and Xu, 2019).

**FIGURE 7:**
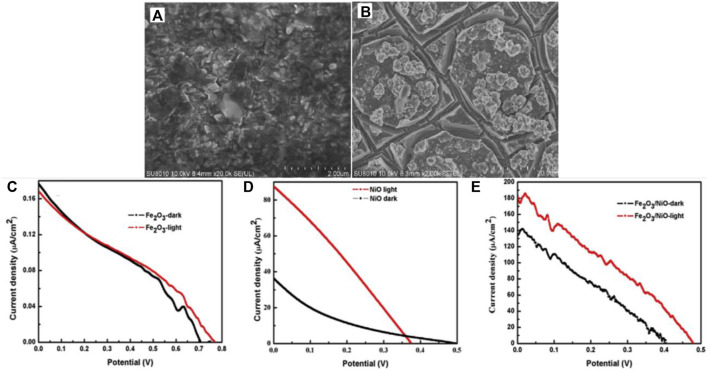
SEM images of **(A)** Fe_2_O_3_ **(B)** Fe_2_O_3_/NiO. **(C–E)** Current density-voltage (I–V) characteristic curves of Fe_2_O_3_, NiO and the heterostructure. Reproduced with permission (Zhang D. et al., 2020), Copyright 2020, Elsevier.

Pt-based cathodes are widely employed in single photoelectrode PFC systems owing to their high activity towards oxygen reduction reaction (ORR). Pt was used in combination with carbon/TiO_2_ as a cathode supported on carbon cloth. (Antoniadou and Lianos, 2009; Sfaelou et al., 2015; Xie and Ouyang, 2017; Xie et al., 2018; Jiao et al., 2019). Pt-decorated Si PVC, Pt modified Buried Junction Silicon (BJS), have also been utilized as cathode, where a Si PVC has been incorporated with Pt cathode for efficient charge generation and transfer (Chen et al., 2014; Xia L. et al., 2016; Zhao et al., 2017; Zeng et al., 2018b). The search for a low-cost alternative for Pt led the researchers towards copper based, carbon-based and graphene-based materials which provide similar electrical conductivity and stability as Pt (Canterino et al., 2009; Sfaelou et al., 2012; Ye et al., 2018; Liu X. H. et al., 2019; Nordin et al., 2019). A recent report analyzed the activity of various cathode materials for PFC. Carbon felt, carbon plate, platinum (Pt)-loaded carbon paper, stainless steel mesh and activated carbon flakes were studied. Due to the high surface area and porous nature, activated carbon flakes showed improved efficiency towards RR120 dye degradation and power generation (Mariaswamy et al., 2020). The reports on PFC, which employs cathode materials other than Pt, have been presented in Table 3 for quick view. Photocathode active PFC utilizing BiOCl as photocathode and Pt as anode with Furfur incorporated as fuel has recently been reported (Wang et al., 2020d). Later the scope of having a p-type photocathode that can act complementary with the n-type photoanode and simultaneously photogenerated charge carriers was widely accepted and studied. The p-type semiconductors with a low bandgap enable the absorption of a wider spectrum extending towards the visible region. Photocathode employed PFCs also have the advantage of better ROS generation and multiple pollutant degradation. Nevertheless, the low stability and constraints of having a more negative Fermi level than photoanode limit the choice of cathode materials. A heterojunction photocathode of n-type Fe_2_O_3_ and p-type NiO has been successfully employed in a recently reported PMFC (Zhang D. et al., 2020) (Figure 7). Oxides and sulfides are the most studied photocathode materials due to their cost effectiveness and ease of synthesis. Cu_2_O is the first reported photocathode material that efficiently absorbed visible light along with WO_3_ photoanode. The credit of enhanced performance of the cell was attributed to mismatched Fermi level positions of WO_3_ and Cu_2_O (Chen et al., 2012). On similar lines, Cu-based oxide materials and their nanostructures have been of great interest to the scientific society due to their Fermi level position, visible light photoactivity and low cost. Cu-based photocathode has been employed in PFC for hydrogen production along with electricity generation and pollutant degradation by Wu et al. (Li J. et al., 2013; Wu et al., 2015). Cu_2_O based heterostructures have also been explored to enhance the charge separation and cathodic activity (Li X. et al., 2018; Lu et al., 2019; Wang et al., 2019).

**TABLE 3:** Quick view on reports of PFC employing cathode materials other than carbon-based materials and Pt.

Anode	Cathode	Electrolyte	Lamp	Fuel	V_oc_ (V)	J_sc_ (mAcm^−2^)	Ref
MTNA	Cu_2_O/Cu	PBS	Solar Simulator	Acetic acid	0.39	0.20	Li et al. (2013a)
WO_3_-TiO_2_ nanotubes	CuO-TiO_2_ nanotubes	Na_2_SO_4_	Visible light	Rh B	0.18	0.04	Yang et al. (2014)
TiO_2_	Ag doped TiO_2_	-	UV-Visible light	Acidic Water	1.59	0.074	Ogura et al. (2014)
TiO_2_ nanorods	C/Cu_2_O/Cu	Na_2_SO_4_	Solar Simulator	Phenol	0.41	0.50	Wu et al. (2015)
BiVO_4_/TiO_2_ nanotubes	ZnO/CuO nanowires	Na_2_SO_4_	Solar Simulator	Glucose	0.53	0.43	Bai et al. (2016)
Ag/AgCl/GO	ZnIn_2_S_4_	Na_2_SO_4_	Visible light	Rh B	0.35	0.52	Liu et al. (2016)
CdS	Porous Ni	Na_2_SO_4_	Visible light	Glucose and Ascorbic Acid	0.571	0.166	Liang et al. (2016)
CdS- TiO_2_	H-NiO_x_ (honeycomb structured)	Na_2_SO_4_-NiCl_2_	Visible light	Bisphenol A	0.85	0.032	Huang et al. (2018)
Polyaniline/TiO_2_ nanotubes	CuO/Co_3_O_4_ nanorods	Na_2_SO_4_	Visible light	Rh B	0.24	0.085	Pan et al. (2018)
TiO_2_ nanotube array	Cu	Na_2_SO_4_	UV lamp	4-chloro-2-methylphenoxyacetic acid (MCPA)	0.24	0.37	Ye et al. (2018)
CdS-ZnS- TiO_2_	CuS/Cu_2_O/Cu nanowire	Na_2_SO_4_	Solar Simulator	KOH + ethanol	1.13	3.18	Wang et al. (2019)
CdS-ZnS- TiO_2_	CuO nanorods	KOH	Solar Simulator	KOH + Methanol	1.0	3.0	Liu et al. (2019a)
g-C_3_N_4_/Fe^0^/TiO_2_	WO_3_	Na_2_SO_4_	Visible light	Berberine Chloride	0.81	2.02	Rabé et al. (2019b)
g-C_3_N_4_/Fe^0^/TiO_2_	WO_3_	Na_2_SO_4_	Visible light	Rh B	0.95	2.32	Rabé et al. (2019a)
TiO_2_	Ag Paste	-	UV lamp	Urea	0.719	-	Qiu et al. (2019)
Ag_3_PO_4_/Fe/GTiP	ZnIn_2_S_4_	-	Visible light	Rh B	0.197	-	Nahyoon et al. (2019a)
Pt	BiOCl	Na_2_SO_4_	Xe lamp	Furfur	-	-	Wang et al. (2020d)
Pt	Fe_2_O_3_/NiO	Na_2_SO_4_	Simulated Solar Light	Methanol	0.48	0.183	Zhang et al. (2020a)

## 4 Developments in applications

**FIGURE 8:**
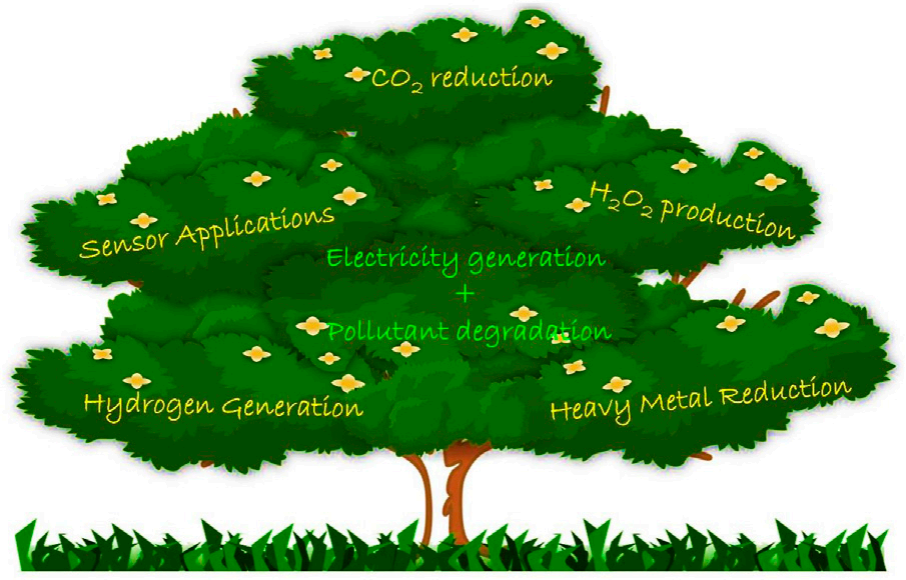
Pictorial representation of applications of PFC.

The majority of the research on PFCs reports the exploration of various electrode materials and design of PFCs. However, the field of application of PFCs remains less explored. In 2005 when Kaneko et al. coined the term Photo Fuel Cell, it was supposed to produce electric power by simultaneously degrading organic pollutants (fuel) under light irradiation. Along with photocurrent generation, hydrogen evolution was also reported (Kaneko et al., 2005). PFCs were also identified with important applications such as hydrogen peroxide generation, CO_2_ reduction, heavy metal reduction and sensor applications, other than electricity generation and wastewater treatment (Figure 8) (Wu et al., 2015; He et al., 2018; Andrade et al., 2019; Du et al., 2019; Jiao et al., 2019; Xiao et al., 2019).

### 4.1 Hydrogen evolution

Generation of electricity along with wastewater treatment can be considered as the primary application of a PFC system. Kaneko et al. and Antoniadou et al. reported the hydrogen evolution from PFC design (Kaneko et al., 2005; Antoniadou et al., 2008). These reports are based on early H_2_ evolution as a result of photoelectrochemical reactions in PFCs. Since then, the major variation in the design of PFCs, capable of producing hydrogen, is the sealed cathode compartment. A process known as “photoelectrochemical reformation” has been used to describe the breakdown of organic materials. In this process, a photocatalyst anode absorbs sunlight to produce electron-hole pairs, which are then used to photoelectrochemically oxidize organic materials by generating holes as shown in Eq. 17 (Patsoura et al., 2007; Chiarello et al., 2009). Through an external circuit, generated electrons are transferred to the cathode where they eventually react with H^+^ to make hydrogen as in Eq. 18.

$$C_{x}H_{y}O_{z}+\left(2x-z\right)H_{2}O\;\to xCO_{2}+\left(4x-2z+y\right)\left(H^{+}+e-\right),\;E^{0}\;varies$$ (17)

$$\left(2x-z+y⁄2\right)\left[2H^{+}+2e^{-}\to \;H_{2}\right],\;E^{0}=0.0\;V\;NHE$$ (18)

The overall reaction:

$$C_{x}H_{y}O_{z}+\left(2x-z\right)H_{2}O\to xCO_{2}+\left(2x-z+y⁄2\right)\;H_{2}$$ (19)

**FIGURE 9:**
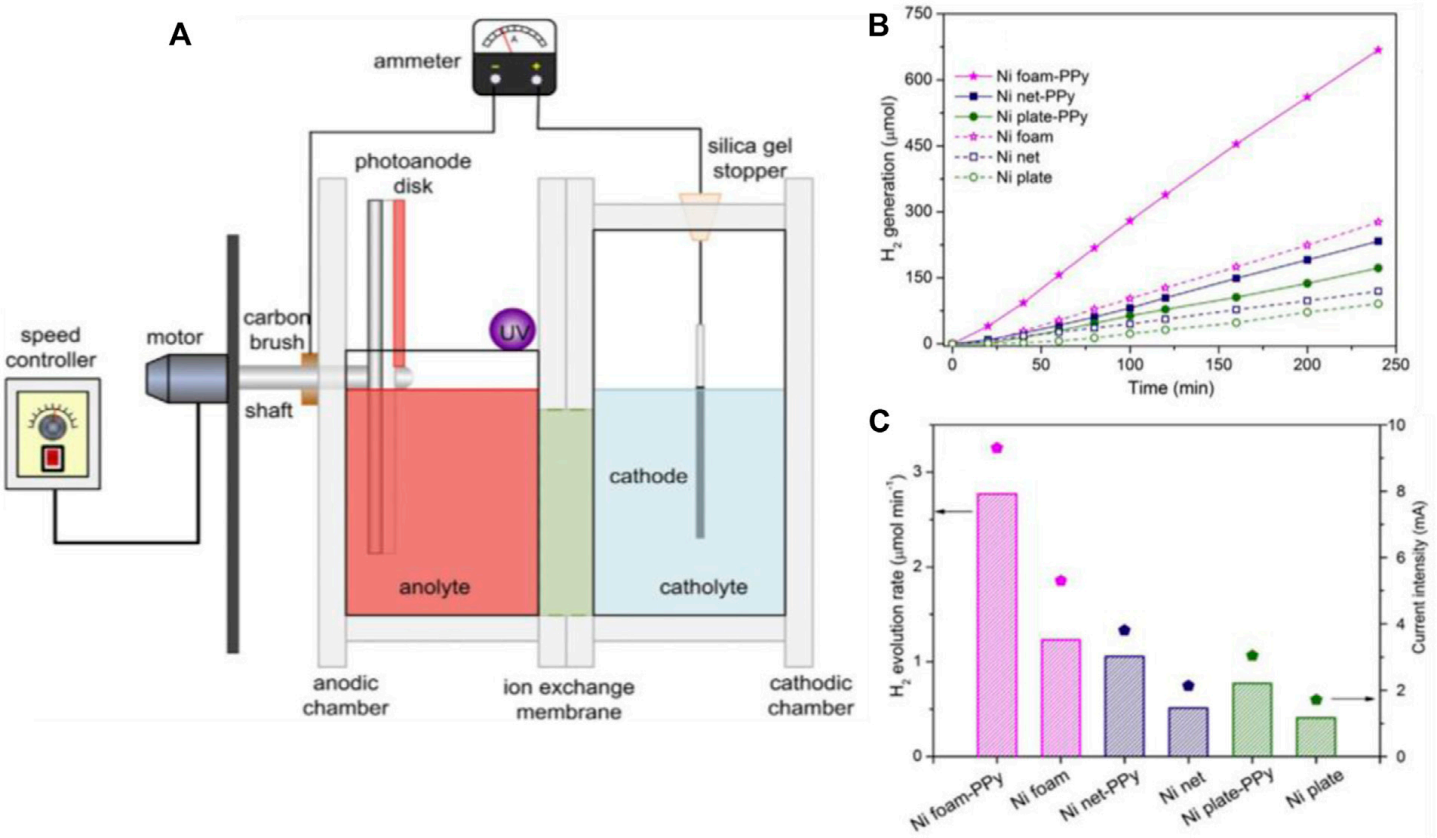
**(A)**Photocatalytic reactor with rotating disc electrode for simultaneous generation of hydrogen and electricity. **(B)** The catalytic evolution of H_2_ by untreated or PPy modified three Nickel substrate structures. **(C)** The H_2_ generation rate and the current flow with several cathodes Reproduced with permission (Tang et al., 2016), Copyright 2016, Elsevier.

The photoelectrochemical reformation may produce usable energy forms like hydrogen and electricity while also cleaning up waste organic material (Strataki and Lianos, 2008; Esposito et al., 2012). Hydrogen evolution was reported in PFC design by providing an electrical biasing (Seger et al., 2012; Liang et al., 2016; Sfaelou et al., 2016). Hydrogen was not catalytically produced in these reports. In other words, electrical biasing was introduced between the electrodes to carry out PEC hydrogen generation. The first report on the simultaneous generation of hydrogen and electricity by utilizing organic pollutants as fuel was by Tang et al., in 2014. In the proposed RDPFC design, TiO_2_ was employed as the photoanode and Pt as the cathode. The bias required for the reaction kinetics was sequestered from chemical bias produced by the difference in charge of electrolytes, NaOH and H_2_SO_4_, anolyte and catholyte, respectively. The group also proposed a Nickel foam-based cathode capable of replacing Pt cathode for catalytic hydrogen generation. Various Ni based substrates has been reported and evaluated for the H_2_ generation and the Ni foam. (Figure 9) (Tang et al., 2014, 2016).

In 2015, we came across the first and only report on a dual photoelectrode PFC that generates hydrogen. It is already known now that in PFC, illumination of semiconductor results in the generation of electrons and holes followed by their separation giving rise to a photovoltage. For n-type semiconductor under light illumination, photo-generated holes are the majority for oxidation. In contrast, for p-type semiconductor under light illumination, photo-generated electrons are the majority for reduction. This photovoltage difference can be exploited for the oxidation of pollutants at photoanode and hydrogen production at photocathode. n-type TiO_2_/CdS/CdSe photoanodes and p-type Cu_2_O were employed to generate sufficient bias based on their difference in Fermi energy levels (Wu et al., 2015). A hybrid system with a combination of PFC-PEC systems has been employed successfully for hydrogen generation by Liu et al. Utilization of TiO_2_ as photoanodes for both cells and Pt on carbon cloth as air-breathing cathode in PFC and Pt as the cathode in PEC (Liu et al., 2017) lead to important findings. However, all the solar light active systems suffer from the challenge of its applicability throughout the day. The complete day operation of PFC can be achieved by the hybrid system design incorporating a mechanism to hold the charge generated during light and then release it in dark. A green supercapacitor assisted photocatalytic fuel cell system with a chemical bias, which aimed for storing photoelectrons by supercapacitors (2 mg cm^−2^) under light irradiation and discharging for hydrogen production under dark to enable continuous hydrogen production has been recently reported. Even though the objective of the whole system was hydrogen evolution even in dark conditions, the team initially failed to achieve it. The group investigated the possible reasons for the failure and arrived on the significance of cathode potential. Cathode potential (-0.25 V vs. Ag/AgCl) was much higher than the onset hydrogen evolution potential (-0.35 V vs. Ag/AgCl) in dark. The system was modified accordingly and a sustainable hydrogen production under illumination (32 μmol L^−1^) and in dark (13 μmol L^−1^), with TiO_2_ photoanode, Ni-PPy cathode was achieved. The report not only emphasis on the sustainable hydrogen generation of a hybrid system, but also conveys the importance of electrode potentials for the hydrogen evolution reactions. (Zhang et al., 2021). The photogenerated electrons in the photoanode are carried to the cathode by the internal bias created by the difference in Fermi levels between the electrodes in a standard PFC. Unfortunately, the bias potential generated by the Fermi level difference is restricted by the photoelectrode’s conduction band position, which is always too small. A hybrid system which can tackle these challenges and efficiently produce hydrogen has been recently reported. The novel HTPFC composed of a front STNR, a rear silicon solar cell (SSC), and a Pt cathode. The STNR and SSC were built as a monolithic photoanode, with the STNR being able to create electron-hole pairs when activated by high energy photons (>2.94 eV). The SSC may be excited by transmission light to provide a bias potential, which will facilitate electron transport from the STNR to the Pt cathode, resulting in hydrogen generation. The system was capable of achieving degradation of TC with a removal ratio of 94.3% after operation for 1.5 h and an average hydrogen generation rate of 28.8 μmolh^−1^cm^−2^. The HTPFC’s higher performance is attributed to its fascinating electrical features, the STNR’s stability, and the rear SSC’s enhanced light exploitation and internal bias (Zeng et al., 2020).

### 4.2 CO_2_ reduction

The ever-increasing energy demand and environmental impact of increasing CO_2_ emission stress the need for a renewable energy source. CO_2_ reduction mechanisms have been considered as a necessity by researchers around the globe. In this regard, efficient CO_2_ reduction mechanisms based on various PFC designs can offer a great deal with simultaneous electricity generation.

In 2014, Morikawa et al. reported a reverse photo fuel cell that employed WO_3_ and layered double halides (LDH) based on Zn, Cu and Ga as photoanode and photocathodes, respectively. The designed system was able to convert CO_2_ to methanol and oxygen, which are the fuels for a conventional fuel cell. The production of fuels as byproducts gained the system the name ‘reverse’ PFC (Morikawa et al., 2014). Recently, PFC designs incorporated with a CO_2_ photoreduction reactor have been reported by Long Jiao et al. and Fengjia Xe et al. TiO_2_ photoanode and Pt cathode have been employed in the PFC. At the same time, TiO_2_ is used in the CO_2_ photoreduction reactor. The integrated design photo reduces CO_2_ as an initial step and produces methanol as a byproduct which acts as fuel for the downstream PFC. In the second step, the methanol is degraded in PFC and electric power is produced. So, on the whole, CO_2_ is photo reduced to produce direct electric power, which addresses the issue of energy demand as well as CO_2_ reduction. In this design, even the unreacted CO_2_ from the upstream photoreduction reactor can be converted to hydrocarbon fuels at photoanode of downstream PFC (Jiao et al., 2019; Xie et al., 2019). An improved photocatalytic reduction of CO_2_ to CH_4_ in a thin-film RDPFC reactor have been recently reported. The yield of CH_4_ in this system was measured to be 7.62 μmol g^−1^ h^−1^, which was achieved by the diffusion of CO_2_ through the thin film and the high solubility of CO_2_ in the aqueous electrolyte solutions. The thin film RDPFC system was capable of a power output of 10.5 μW cm^−2^. The improved efficacy of the system is attributed towards the thin film RDPFC design which incorporated a TiO_2_– NT rotating disc photoanode (Zhang et al., 2020c).

**FIGURE 10:**
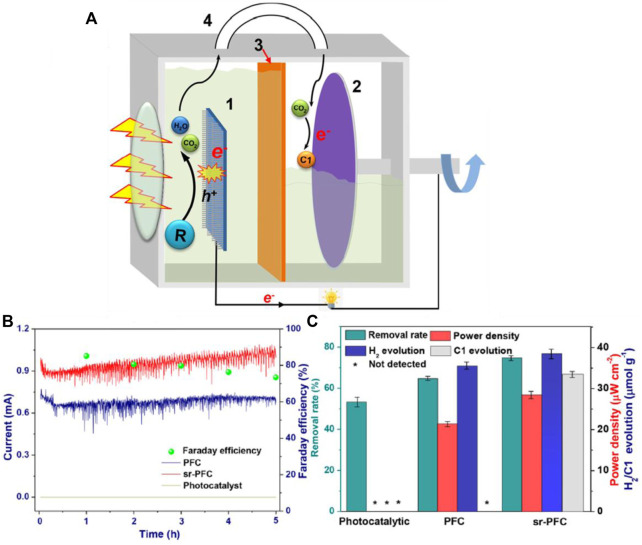
**(A)** Schematic diagram of the experimental setup. 1. TiO_2_-NT photoanode; 2. Ti_3_C_2_ cathode; 3. Cation-exchange membrane; and 4. Gas channel. **(B)** Current response and Faraday efficiency of CO_2_ reduction **(C)** by-product analysis in different operating system within 5 h. Reproduced with permission (Zhang et al., 2020b), Copyright 2020, American Chemical Society.

A CO_2_ self-driving and self-recycling photocatalytic fuel cell (PFC) system was recently reported by Zhang et al. Comparing the carbon neutral PFC system to the traditional photocatalytic system, the PFC system increased organics removal by 40% and added 6.7 μmolg^−1^h^−1^ additional C1 fuel yield rates. The CO_2_ generated at anode upon wastewater degradation was continuously supplied to rotating cathode (Figure 10). The CO_2_ reduction reaction occurring at the cathode accelerated the electron−hole separation in photoanode and facilitated the formation of C1 fuel in the cathode (Zhang et al., 2020b). A recent report which investigated the role of CO_2_ in the photocatalytic fuel cell drew many interesting scientific conclusions. The PFC system with TiO_2_ as the photoanode and methanol as a fuel under acid condition was tested by making the anode electrolyte saturated with CO_2_. It was revealed that the performance of the photocatalytic fuel cell can be improved at low fuel concentration by the presence of CO_2_. When there was no CO_2_ in the anode electrolyte, the OCV was 0.97 V, the SCC was 0.107 mA cm^−2^, the MPD was 0.052 mW cm^−2^. But, when the anode electrolyte was saturated with CO_2_, although the OCV was close to the case without CO_2_, the SCC and MPD were 0.128 mA cm^−2^ and 0.072 mW cm^−2^, respectively, both of which were higher than the case without CO_2_. These results deny the conventional wisdom that CO_2_ as a product should be removed as soon as possible to improve the performance. Youxu et al. also discusses about the plausible reasons towards the interesting phenomena they observed and those reasons are as follows: TiO_2_ had an adequate conduction band potential of -0.2 V vs. NHE, it was more negative than the thermodynamic potential for CO_2_ reduction to methanol at pH = 1 (-0.026 V vs. NHE), which will facilitate the photocatalytic reduction of CO_2_ to organics at the photoanode, which increases the methanol concentration at the catalytic surface to alleviate the mass transfer issue encountered in the low methanol concentration operation, and hinders the recombination of the electron-hole pairs (Yu et al., 2021).

### 4.3 Hydrogen peroxide production

Hydrogen peroxide finds its applicability in multiple areas of pharmaceuticals, advanced oxidation processes, Fenton reactions and even as energy storage. It is considered to store chemical energy while being used in hydrogen peroxide fuel cells and even as an oxidant in many other types of fuel cells. Many methods have been adopted for efficient H_2_O_2_ production like the anthraquinone oxidation method, electrochemical advanced oxidation technologies and microbial fuel cells. However, the issues related to potential toxicity, high operational costs, the expense of external power supply, complex operation, stringent working conditions and long start-up time due to the presence of bacteria have suppressed the large-scale applications of these technologies.

**FIGURE 11:**
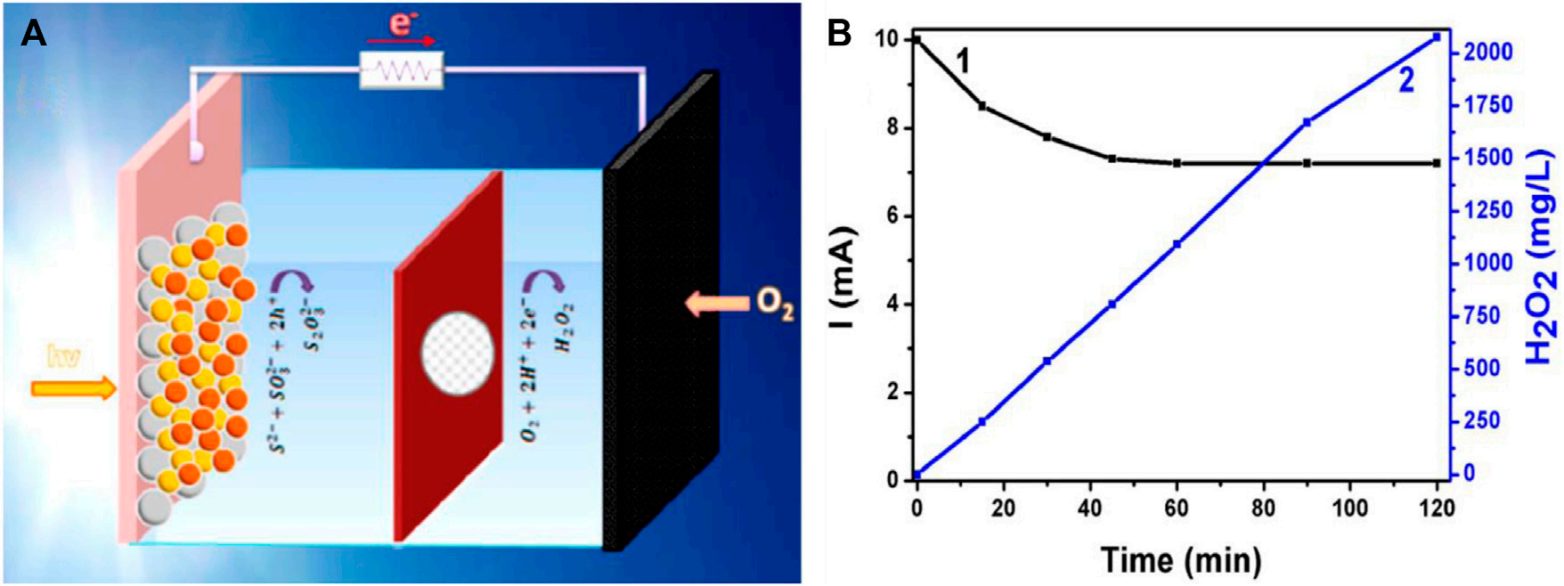
**(A)** Schematic design of the photofuel cell reactor used for H_2_O_2_ production. **(B)**Evolution of the short-circuit current (1) and of the quantity of photoelectrochemically produced H_2_O_2_ (2) using a PFC operated with a ternary semiconductor photoanode. Reproduced with permission (Andrade et al., 2019), Copyright 2019, Multidisciplinary Digital Publishing Institute.

PFC as a promising technology for efficient H_2_O_2_ production has been reported by a few groups recently. Santos Andrade et al. reported two PFC systems with CdS/TiO_2_ and CdSe/CdS/TiO_2_ as visible light active photoanodes, respectively and carbon black/carbon cloth as the cathode (Figure 11). NaHCO_3_ was used as a catholyte which enhanced the water oxidation by HCO_3_^−^ catalysis and enhanced the H_2_O_2_ production in the cathode (Andrade et al., 2019).

$$O_{2}+2H^{+}+2e^{-}\to H_{2}O_{2}$$ (20)

A novel mechanism integrating PFC and Fenton reaction assisted by reverse electrodialysis (PREC) has been recently proposed by Peng Xu et al. Reverse electrodialysis (RED) is a novel technology for electricity production. Alternately stacked anion exchange membranes (AEM) and cation exchange membranes (CEM) separated by high concentration (HC) and low concentration (LC) cells are collectively called RED stack. When the stack is supplied with suitable HC and LC solutions, current is generated due to the migration of ions through AEM and CEM. In the PREC reports, ZnO/Zn is used as photoanode and FeVO_4_/carbon felt (CF) is used as cathode. The photogenerated electrons from the photoanode separate and travel efficiently towards the cathode with an enhanced bias of RED stack and hydrogen peroxide is produced at the cathode by the two-electron reduction of dissolved O_2_ on the FeVO_4_/CF surface (Xu et al., 2019b; Xu and Xu, 2019). A PFC system where the *in-situ* generation of H_2_O_2_ is reported, but the generated H_2_O_2_ is consumed by the system itself as a source of hydroxyl radical and superoxide radical. The generation of radicals in turn improves the overall efficacy of the system. PFC system was comprised of SSC incorporated Fe_2_O_3_/BiVO_4_ photoanode and graphene felt cathode. The electron-hole pairs are generated upon light irradiation in photoanode and the photogenerated holes oxidize H_2_O to oxygen and hydroxyl radical owing to its potential. SSC acts as an additional potential supplier in the system. Electrons reaching the graphene felt cathode combines with adsorbed oxygen to produce H_2_O_2._ Due to its high hydrophilicity, generated H_2_O_2_ is quickly transferred to the solution for radical chain reaction with added Fe ions in the system (Li et al., 2020). A record-high H_2_O_2_ concentration of 110 mmol L^−1^ is achieved by Wenjun Fan et al., where the group have employed polyterthiophene (pTTh), a metal free narrow-bandgap polymeric semiconductor, as cathode is an efficient photocathode for H_2_O_2_ production in alkaline solution (Fan et al., 2020). A work has studied the photoelectrochemical production of hydrogen peroxide and the direct use of the produced material for the degradation (decolorization) of three common dyes: methylene blue, basic blue 41 and acid orange 7. The reported PFC operated with a CdS-sensitized mesoporous titania photoanode and a simple carbon cloth cathode carrying a hydrophobic layer of mesoporous carbon (carbon black). Hydrogen peroxide was produced in the cathode compartment of the PFC by atmospheric oxygen reduction, with a very high Faradic efficiency nearing 100%. The system was highly effective since hydrogen peroxide production rate was close to the theoretically expected value. The maximum quantity of H_2_O_2_ produced in the present case after 4 h of continuous operation was 1,200 mg L^−1^ which corresponds to about 35 mmol L^−1^. These results open a route for the conversion and storage of solar radiation in the form of chemical energy (Dhawle et al., 2021).

### 4.4 Heavy metal reduction

**FIGURE 12:**
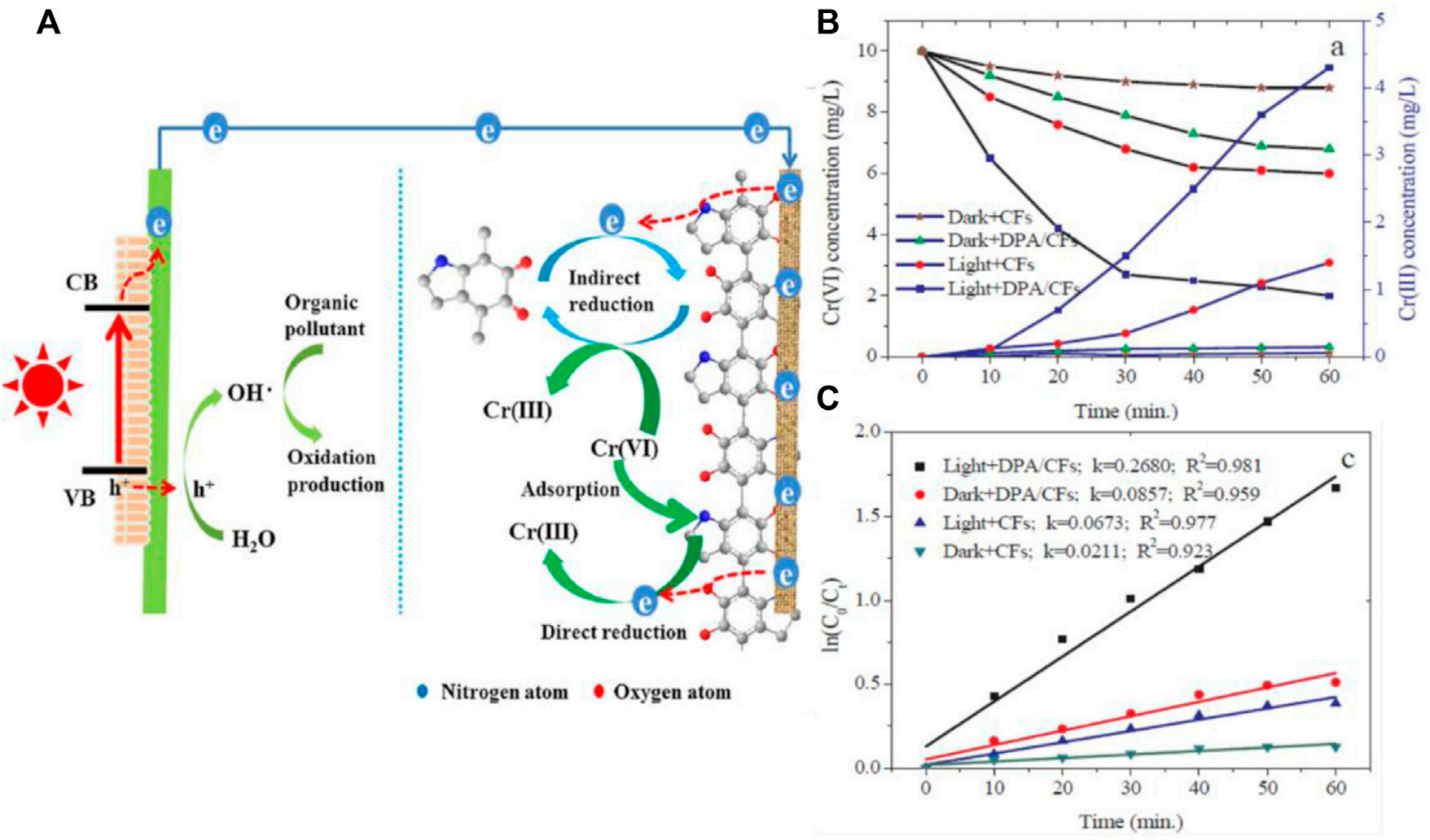
**(A)** Schematic illustration depicting the simultaneous degradation of Bisphenol A and Cr (VI) reduction. **(B)** The concentrations of Cr (VI) and Cr (III) in the cathode chamber. **(C)** The Cr(VI) reduction kinetics with the carbon felt (CF) and carbon felt cathode modified by dopamine (DPA/CF). Reproduced with permission (Xiao et al., 2019)Copyright 2019, Elsevier.

Wastewater treatment has been a hot topic for many years for the scientific world due to its crucial role in environmental reconditioning. We have numerous reports based on biological treatment, membrane technology, chemical and electrochemical techniques that can effectively remove pollutants. However, these techniques completely ignore the ways of energy recovery from wastewater. The PFC designs find their importance in the area where energy recovery and pollutant degradation are steered simultaneously. The presence of heavy metals in water has been realized as a huge problem, related to the fact that removal of heavy metals is a tedious task. Most of the heavy metal reduction techniques ignore the chemical energy which can be recovered from the same. Recently Xiao et al. reported a PFC which can simultaneously degrade Bisphenol A and reduce Cr (VI) along with electricity generation. The report is considered to be the first report on using dopamine modified carbon as cathode and BiVO_4_ as photoanode for heavy metal reduction using PFC. As BPA gets photocatalytically degraded in anode chamber by BiVO_4_, Cr (VI) gets reduced to less mobile and less toxic Cr (III) in the cathode chamber. The schematic illustration of the PFC working is as shown in Figure 12. Even though a PFC design is used, the reports do not state that the heavy metal reduction is purely based on the photocatalytic activity. Adsorption property and surface properties of cathode plays a vital role in reduction mechanism (Xiao et al., 2019). A complete multifunctional PFC which can degrade pollutants, reduce heavy metal and generate power was introduced later by Liu et al. The device degraded model pollutant MB and reduced Cr (VI) with an enhanced power generation.

TiO_2_ and graphite were employed as photoanode and cathode, respectively, in separate chambers. Complete degradation of MB and 96.8% removal of Cr (VI) with a cathodic efficiency of 95.1% was achieved, with a maximum power output being 1 W m^−2^. Cr(VI) reduction is thermodynamically more advantageous than that of oxygen, and it could draw more photogenerated electrons to the cathode to thus render a higher electric power, better degradation and enhanced removal percentage (Liu X. H. et al., 2019). A synergetic photocatalytic degradation of organic dye (X3B) in anode and the reduction of heavy metal Cr(VI) in cathode are carried out in an improved dual-chamber PFC device with TiO_2_ and core–shell structured Ag@Fe_2_O_3_ nanoparticles as photoanode and cathode materials in a recent report. Ag@Fe_2_O_3_ shows the highest catalytic activity to the reduction of Cr (VI) with an apparent kinetic constant of 0.058 min^−1^, even much better than commercial Pt foil. It is worth noting that the *k*_*obs*_ value of Fe_2_O_3_ is only lower than that of Ag@Fe_2_O_3_ but much higher than those two noble metals (Pt and Ag), indicating that Fe_2_O_3_ truly has a considerable catalytic ability for the reduction of Cr (VI). The improved performance in cathodic reduction of Cr(VI) with Ag@Fe_2_O_3_ cathode can be attributed to directional transport channels supplied by Ag cores and desirable catalytic activities of Fe_2_O_3_ shells (Sun et al., 2022).

### 4.5 Sensor applications

Photocatalytic fuel cell-based sensors are a very recent advancement in the field of applications of PFC. The first report on PFC based sensor was published in 2016 by Kai Yan et al. The reported self-powered sensor consisted of Ni (OH)_2_/CdS/TiO_2_ photoanode and hemin graphene cathode. The group successfully developed a glucose sensor with a detection limit as low as 5.3 µM. The H_2_O_2_ reduction at the cathode and the visible light activity made the sensor a significant milestone in the application pathway of PFC (Yan et al., 2016).

One of the major drawbacks in combining the practicality of photocatalysis in sensor platforms is the non-selective nature of the process. Hence the same group came up with a PFC sensor, which incorporated a molecularly imprinted polymer (MIP) for the selective sensing of p-Nitrophenol. Graphene modified GCE was employed as the anode, which oxidised the Ascorbic acid and PbS quantum dot modified GCE was used as the photocathode, which reduced the p-NP under irradiation. The PFC-based MIP sensor achieved a p-NP detection limit of 0.031 µM (Yan et al., 2017). MIP integrated PFC based novel self-powered biosensor was constructed to achieve sensitive and specific detection of aflatoxin B1 (AFB1). MoS_2_–Ti_3_C_2_T_x_MXene (MoS_2_–MX) served as the photoanode material for the first time by forming a heterojunction structure, which can enhance the photocurrent by about 3-fold and greatly improve the photoelectric conversion efficiency. The self-powered biosensor showed a wide dynamic range of 0.01–1,000 ng ml^−1^ with a detection limit of 0.73 pg ml^−1^ with MIP and PFC combined to act as recognition and signal conversion elements respectively (Jin et al., 2021). A work which utilises the material property of Ni-based materials to form Ni^2+^/Ni^3+^redox couple for glucose detection. Dual system based on PEC and PFC techniques was studied by utilising Ni(OH)_2_/TiO_2_ as anode and Pt cathode, with a detection limit of glucose as low as 5 µM (Yang et al., 2017). Later in 2018 aptamer-based PFC sensing application was reported by Kai Yan et al. A visible, membrane less, H_2_O_2_ PFC system was developed by the group for sensing PCB_77_, which potentially causes cancer and many adverse health issues. Au nanoparticles decorated g-C_3_N_4_ nanosheet and hemin graphene nanocomposites were used as photoanode and cathode, respectively. Binding aptamer (5′-SH-(CH_2_)_6_-GGC GGG GCT) immobilised on photoanode couples with PCB_77_ present in the solution and affects the electron transfer, which in turn enhances the sensitivity and selectivity of the system. The same group came up with MIP based sensor for Bisphenol-A sensing with MIP/g-C_3_N_4_ on FTO as anode and Pt cathode. The binding property of MIP and efficient photocatalytic activity of carbon nitride synergistically brought down the detection limit of BPA to 1.3 µM (Yan et al., 2018b; 2018a). Few reports on PFC sensors for enhanced detection of pharmaceuticals and enzymes (affecting both humans and animals) were reported in 2019. The first report on a dual photoelectrochemical-PFC sensor for sensing of microcystin-LR, a potential toxin produced by cyanobacteria present in drinking water, was proposed by Du et al. This system had a detection limit of 0.67p.m. and employed TiO_2_ nanoparticles and nitrogen-doped graphene with BiOBr as anode and cathode respectively (Du et al., 2019). Kanamycin, an antibiotic that causes side effects related to hearing and balance problems and can even cause kidney-related health issues, was efficiently detected using an aptamer-based PFC sensor, which employed graphene doped BiVO_4_ as photoanode and Pt as the cathode. Aptamer-based sensing helped in selectivity and a detection limit of 1 nM was achieved (Ji et al., 2019). A similar antibiotic, Bleomycin, which possesses severe concomitant effects related to lungs, was detected using a visible light activated PFC sensor by Mengjun et al. Aptamer modified CdS-In_2_S_3_ and Pt was utilised as photoanode and cathode for the efficient sensing with a detection limit of 1 nM(Sun et al., 2019).

**FIGURE 13:**
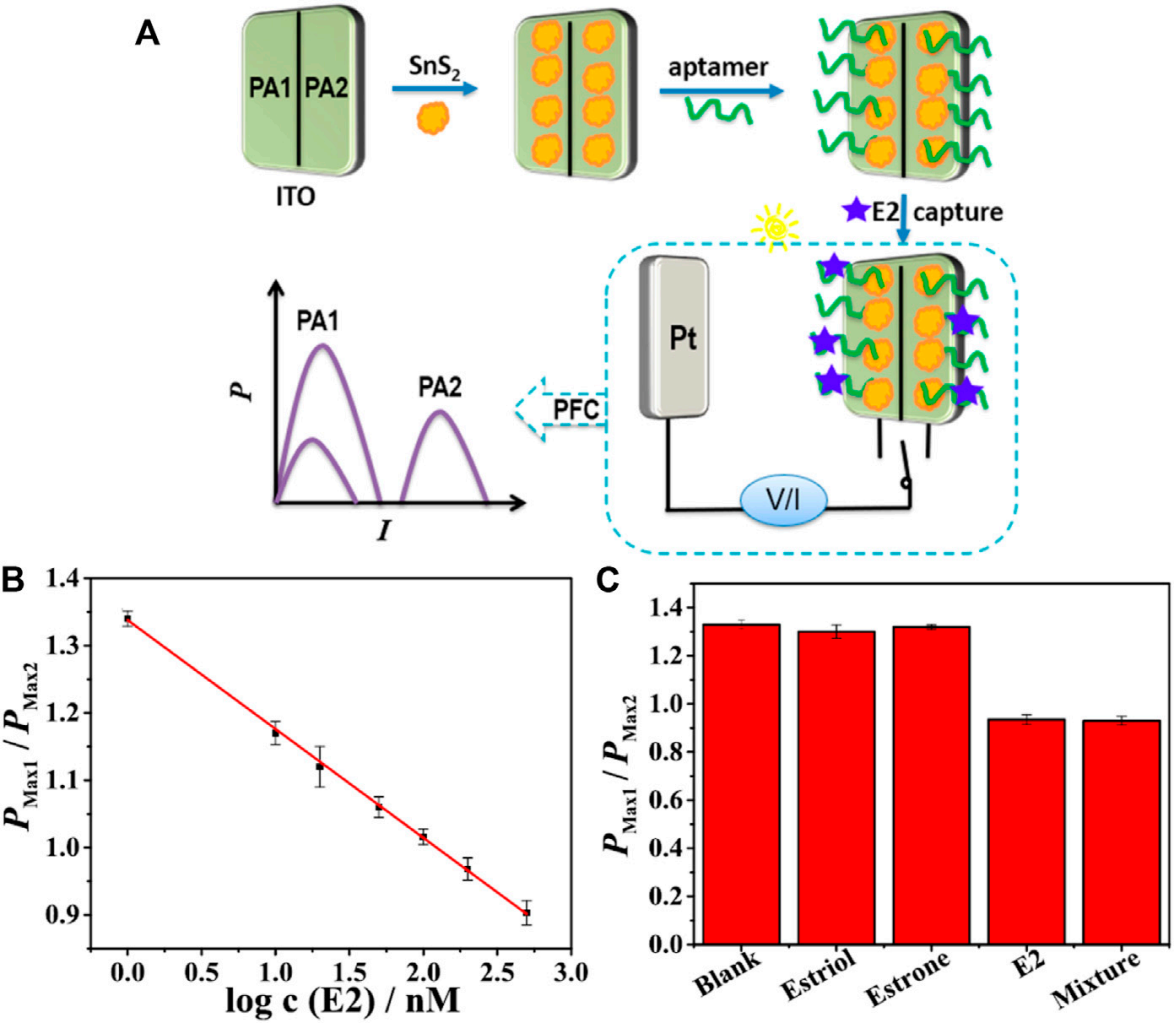
**(A)** Schematic illustration of fabrication process of dual-channel PFC for ratiometric self-powered aptasensing**(B)** Calibration curve for E2 detection by the proposed ratiometric self-powered aptasensor based on dual photoanodes. **(C)**Histogram for P_max1_/P_max2_ values of the proposed sensor toward 200 nM estriol, estrone, E2, and their mixture. Reproduced with permission (Yao et al., 2020), Copyright 2020, American Chemical Society.

Recently, a Tyrosinase PFC sensor which finds its application in melanoma diagnosis was developed by Kai Yan et al. with a detection limit as low as 0.005 U mL^−1^ g-C_3_N_4_/Bi_2_S_3_ and hemin graphene was used as photoanode and cathode, respectively (Yan et al., 2019). A photoelectrochemical (PEC) oxidation of glucose at tungsten trioxide nanoplate (WO_3_ NP) electrode for glucose sensing application and photo fuel cell (PFC) application has been reported recently. The WO_3_-based PEC sensor shows high sensitivity of 68.15 μA cm^−2^ mM^−1^ in glucose detection for the concentrations in the range of 0.1∼0.5 mM. Moreover, electricity can be extracted from photo-oxidizing of glucose on WO_3_ NP electrode in a PFC device and the output strongly depends on the concentration of glucose fuel (Zhang B. et al., 2019). A Fe_2_O_3_ nanorod-based PFC sensor, which shows excellent performance in glucose detection for the concentrations in the range of 0.5–2.5 mM with a sensitivity of 3.53 μAcm^−2^mM^−1^. The report not only realizes the electricity generation from renewable solar energy and bioenergy but also accomplishes the detection of glucose by self-power (He et al., 2020). The self-powered PFC sensors reported so far faces the challenge of varying intensity of light source, which will affect the sensing parameters and the outputs. A ratiometric PFC design, which is capable of addressing the flux variations on incident light was reported by Xiaoling Yao et al. (Figure 13). A highly selective detection of 17β-estradiol (E2) is realised, based on a dual-channel PFC constructed with two photoanodes which could effectively avoid the fluctuation of the light intensity. E2 was quantified *via* the ratio of output power density values from dual photoanodes. The sensing signal was linearly related to the logarithm of E2 concentration in the range of 1–500 nM, with a detection limit (3S/N) of 0.12 nM (Yao et al., 2020). The development of PFC self-powered sensors from model analyte sensing to various pollutants detection in a short period of time projects its potential to become the simplest and self-powered replacement for conventional sensors.

## 5 Conclusion and future perspectives

A chronological growth of Photocatalytic Fuel Cells on the grounds of system design, materials used and potential applications has been consolidated in the article. The design of the PFC system has been discussed as SCSP design, DCSP design, SCDP design, Triple chamber system, MFC design, RDPFC system and Hybrid systems based on the number of chambers, characteristics of the electrodes and interaction of the overall processes. Single Chamber Single Photoelectrode system was the design of the first ever reported PFC system. It still stands out as the most explored design accounting to its simplicity in design and efficient charge transfer properties. In the case of SCSP, the simplicity in design also points towards the narrowing of applicability in various areas. The upcoming designs of dual chamber PFC systems shed light towards a bright horizon of multiple applications on a single system such as simultaneous degradation of various dyes in the anodic and cathodic chamber, degradation and heavy metal reduction simultaneously in each chamber, degradation in one and hydrogen evolution in the other. Triple chamber systems which work on SGP, RDPFCs, which incorporate photocatalysis along with properties of thin film surfaces and MFCs that rely on microscale optofluidics are promising system designs that enjoy great room for further exploration of scientific society. Hybrid systems can be stated as the future of PFCs, where various well-explored systems and PFC are combined. Fenton-PFC systems, SMPP systems, PFC-PEC systems, PFC-PVC systems, CO_2_ reduction-PFC systems, Micro supercapacitor-PFC systems and RED-PFC systems have been employed for various applications ranging from degradation along with electricity generation to H_2_O_2_ production, TOC and total nitrogen removal and even on the grounds of energy storage. Further, the reports on novel designs of PFC primarily emphasize its novelty and enhancing the efficiency of the PFC system. In our view, the novel designs from research groups should pay attention to exploiting the arena of various applications rather than merely focusing on enhancing primary PFC mechanisms. Even though design plays a vital role in the efficiency of the PFC system, a proper selection of materials also contributes equally towards the overall performance of the system.

Anode and cathode material properties play a prominent role in the efficiency of the system. TiO_2_ can still be considered as the champion material as photoanode and Pt has been widely employed as the cathode. Nanostructures of TiO_2_ and narrow bandgap material-TiO_2_ heterostructures have proven to be great anode candidates with their efficient electron transfer and wider absorption spectrum. ZnO, BiVO_4_, WO_3,_ Fe_2_O_3_ have also shown better charge separation and transfer. Meanwhile, cathode materials lack selection choices due to the need for better electronic conductivity, stability and corrosion resistance in photoelectrode systems and suitable band edge positions as that of the anode in dual photoelectrode systems. Carbon-based materials were found to possess properties that could allow them to be considered an optimum replacement for costly Pt electrodes. As dual photoelectrodes system came into the scene, the cathode research also acquired a velocity and many p-type semiconductor materials such as sulfides, Cu-based materials, spinel-ferrite structures have been studied so far. Even though there are numerous reports on various materials other than TiO_2_ and Pt, the research on materials implemented for electrodes still has to be considered insufficient when compared with the booming material research in the photocatalysis regime. The scope of exploring materials and their unique properties for PFC research is a great arena for researchers around the globe.

PFC was initially employed as a system for simultaneous pollutant degradation and an electric power source. As the timeline progressed, the arena of application widened and has since been extended towards H_2_ and H_2_O_2_ generation, CO_2_ and heavy metal reduction and sensor applications. The environmental importance of PFC is understood when we realize that all these applications exist along with pollutant degradation and electricity generation. Even though the concept has immense potential in various applications, the scientific community should emphasize on the utilization of natural solar irradiation so that a much sustainable technology can be established. From the above discussion the key take home points can be consolidated as 1) Hybrid PFCs are the future of PFCs and the scientific community should be focusing more on the synergy of PFCs along with other potential systems such as Fenton systems, photovoltaic systems *etc.* 2) The photoelectrode material research should be more concentrated on low cost, highly efficient materials and a proper focus has to be placed on the visible light active photoelectrodes and p-type photocathodes 3) The energy consuming light source such as UV-light, Visible lamp, solar simulator *etc.* should be used as a preliminary approach towards PFCs and the ultimate objective should be a system which is active under natural solar irradiation, which will substantiate the fundamental idea of PFC research. Proper selection of design and materials play a vital role in applications and focusing on those that can deliver high impact works on the applications of PFC.

## Glossary

AMSC—: asymmetric micro super capacitor
AEM—: anode exchange membrane
BJS—: buried iunction silicon
BPA—: bisphenol A
BNC—: biomass derived N-doped carbon
CEM—: cathode exchange membrane
DCSP—: dual chamber single photoelectrode
EF—: electro-fenton
HC—: high concentration
LDH—: layered double halides
LC—: low concentration
MIP—: molecularly imprinted polymer
MPD—: maximum power density
MFC—: micro fuel cell
OCV—: open circuit voltage
ORR—: oxygen reduction reaction
PEM—: proton exchange membrane
PVC—: photovoltaic cell
PFC—: photocatalytic fuel cell
PEC—: photoelectrochemical cell
RDPFC—: rotating disc photocatalytic fuel cell
Rh B—: rhodamine B
RED—: reverse electro dialysis
SCSP—: single chamber single photoelectrode
SCDP—: single chamber dual photoelectrode
SGP—: salinity gradient power
SMPP—: self-sustaining monolithic photocatalytic/photovoltaic system
SCC—: short circuit current
STNR—: silicon doped TiO_2_ nano rod array
SSC—: silicon solar cell
TOC—: total organic carbon
